# Virus‐Induced Cellular Senescence Causes Pulmonary Sequelae Post‐Influenza Infection

**DOI:** 10.1111/acel.70140

**Published:** 2025-06-20

**Authors:** Larissa Lipskaia, Lou Delval, Valentin Sencio, Emmanuelle Born, Séverine Heumel, Amal Houssaini, Clara Valentin, Stefano Raviola, Lucie Deruyter, Fabiola Silva Angulo, Vincent Gros, Elisabeth Marcos, Shariq Abid, Mira Goekyildirim, Vanessa Contreras, Jean Michel Flaman, Roger Le Grand, Ronan Le Goffic, David Bernard, Serge Adnot, François Trottein

**Affiliations:** ^1^ Institut Mondor de Recherche Biomédicale (IMRB), FHU SENEC Univ. Paris‐Est Créteil, INSERM U955 Créteil France; ^2^ Département de Physiologie‐Explorations Fonctionnelles and FHU SENEC Hôpital Henri Mondor, AP‐HP Créteil France; ^3^ U1019—UMR 9017—CIIL—Center for Infection and Immunity of Lille Univ. Lille, CNRS, INSERM, CHU Lille, Institut Pasteur de Lille Lille France; ^4^ Institute for Lung Health Justus Liebig University Giessen Germany; ^5^ Center for Immunology of Viral, Auto‐Immune, Hematological and Bacterial Diseases (IMVA‐HB/IDMIT) Université Paris‐Saclay, Inserm, CEA Fontenay‐aux‐Roses France; ^6^ Equipe Labellisée la Ligue Contre le Cancer, Centre de Recherche en Cancérologie de Lyon, Inserm U1052, CNRS UMR 5286, Centre Léon Bérard Université de Lyon Lyon France; ^7^ Institut National de Recherche Pour l'Agriculture, l'Alimentation et l'Environnement UR892, UVSQ, VIM Université Paris‐Saclay Jouy‐en‐Josas France

**Keywords:** acute response, cellular senescence, chronic damage, epithelial damage, fibrosis, genetic approach, influenza, senolysis

## Abstract

Influenza A virus (IAV) infection causes acute and long‐term lung damage. Here, we used immunostaining, genetic, and pharmacological approaches to determine whether IAV‐induced cellular senescence causes prolonged alterations in lungs. Mice infected with a sublethal dose of H1N1p2009 exhibited cellular senescence, as evidenced by increased pulmonary expression of p16, p21, β‐galactosidase and the DNA damage marker gamma‐H2A.X. Cellular senescence began 4 days post‐infection (dpi) in the bronchial epithelium, then spread to the lung parenchyma by 7 and 28 dpi (long after viral clearance), and then declined by 90 dpi. At 28 dpi, the lungs showed severe remodeling with structural bronchial and alveolar lesions, abrasion of the airway epithelium, and pulmonary emphysema and fibrotic lesions that persisted up to 90 dpi. In mice and nonhuman primates, persistence of senescent cells in the bronchial wall on 28 dpi was associated with abrasion of the airway epithelium. In p16‐ATTAC mice, depletion of p16‐expressing cells with AP20187 reduced pulmonary emphysema and fibrosis and led to complete recovery of the airway epithelium at 28 dpi, indicating a marked acceleration of the epithelial repair process. Treatment with the senolytic drug ABT‐263 also accelerated epithelial repair without affecting pulmonary fibrosis or emphysema. These positive effects occurred independently of viral clearance and lung inflammation at 7 dpi. Finally, AP20187 treatment of p16‐ATTAC mice at 15 dpi led to complete recovery of the airway epithelium at 28 dpi. Thus, virus‐induced senescent cells contribute to the pulmonary sequelae of influenza; targeting senescent cells may represent a new preventive therapeutic option.

## Graphical abstract

Genetic or pharmacological depletion of senescent cells during influenza A virus infection led to complete recovery of the airway epithelium, indicating a marked acceleration of the epithelial repair process. Targeting senescent cells may represent a new preventive therapeutic option to reduce post‐influenza chronic lung damage.

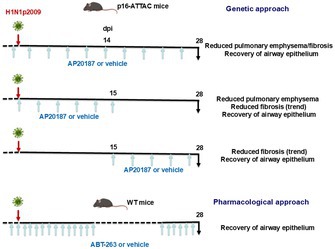

**Abbreviations**
Bcl‐2: B cell lymphoma‐2
COVID19: coronavirus disease 19
dpi: day post‐infection
IAV: influenza A virus
SARS‐CoV‐2: severe acute respiratory syndrome coronavirus 2
SASP: senescence‐associated secretory phenotype

## 1 Introduction

Respiratory viral infections raise serious health and economic issues worldwide, as exemplified by the recent coronavirus disease 2019 (COVID‐19) pandemics. Influenza A virus (IAV) infections also cause substantial morbidity and mortality worldwide, despite the implementation of vaccination programs and antiviral treatment (Dunning et al. 2020). Respiratory viruses replicate in the upper and lower respiratory tracts and thereby cause bronchitis and bronchiolitis, diffuse alveolar damage, and interstitial and airspace inflammation. Although the replication rate of RNA viruses like IAV peaks 2–4 days after infection and declines thereafter, damage to the respiratory epithelium and lung parenchyma persists longer and can result in major adverse events and sequelae (Umeda et al. 2010; Herold et al. 2015; Narasimhan et al. 2024). These events include an elevated risk of secondary infection with bacterial pathogens, exacerbations of chronic obstructive pulmonary disease in children and adults, and the development or worsening of idiopathic pulmonary fibrosis or lung emphysema in a substantial proportion of infected people (Sheng et al. 2020). Thus, by triggering the initiation, exacerbation or progression of chronic lung diseases, IAV infection exerts well‐characterized, long‐term, harmful effects. However, the details of the link between infection and chronic disease remain unclear. Here, we hypothesized that cellular senescence has a role in this setting.

Cellular senescence occurs when cells are exposed to an acute and/or chronic stressor (for review, see Gorgoulis et al. 2019). These stressors are very diverse and include DNA damage, replicative exhaustion, oxidative or metabolic stress, oncogene expression, and inflammation. Cellular senescence consists of (i) resistance to apoptosis and (ii) a stable arrest in proliferation. The key mediators of the cell cycle arrest include the cyclin‐dependent kinase inhibitors p21Cip1 (referred to hereafter as p21) and p16INK4A (hereafter p16) (He and Sharpless 2017). Another important feature of senescent cells is the acquisition of a specific senescence‐associated secretory phenotype (SASP), characterized by the release of inflammatory cytokines, immune modulators, proteases, and various effectors that reinforce senescent phenotypes and alter tissue microenvironments (He and Sharpless 2017; Gorgoulis et al. 2019; Childs et al. 2017). Senescent cells accumulate with age; this phenomenon is due in part to the failure of the immune system to remove them. The chronic accrual of senescent cells in tissues is a key process in age‐related diseases, including lung diseases such as pulmonary lung fibrosis and chronic pulmonary disease (Barnes et al. 2019; Yao et al. 2021). In recent years, the notions whereby (i) a (respiratory) viral infection can cause (lung) cell senescence and (ii) infection‐ or aging‐induced cellular senescence has an impact on disease severity have emerged (Lee et al. 2021; Camell et al. 2021; Oikawa et al. 1987; Lipskaia et al. 2022; Evangelou et al. 2022; Tsuji et al. 2022; Delval et al. 2023a; Aguado et al. 2023). Although influenza is associated with the accumulation of senescent cells in lungs (Kulkarni et al. 2019; Lv et al. 2022; Schulz et al. 2023), the consequences on long‐term lung disease have not yet been studied. In the present study, we postulated that the persistence of lung senescent cells impedes lung repair and underlies the development of chronic impairments of the lung (e.g., fibrosis and emphysema). To this end, we first monitored the temporal and topographic relationships between senescent cell accumulation and pulmonary lesions within 3 months of administration of a sublethal dose of IAV H1N1p2009 in mice. Next, we determined whether the removal of senescent cells would improve lung recovery following an IAV infection, accelerate the repair of the airway and alveolar epithelium, and thereby protect against the development of post‐viral lung diseases. Our study shows that cellular senescence contributes to long‐term pulmonary sequelae after primary influenza pneumonia thereby establishing a link between IAV‐induced prolonged pulmonary damage and cell senescence.

## 2 Results

### 2.1 Influenza Causes Long‐Lasting Lung Damage Concomitant With the Persistence of Senescent Cells

**FIGURE 1:**
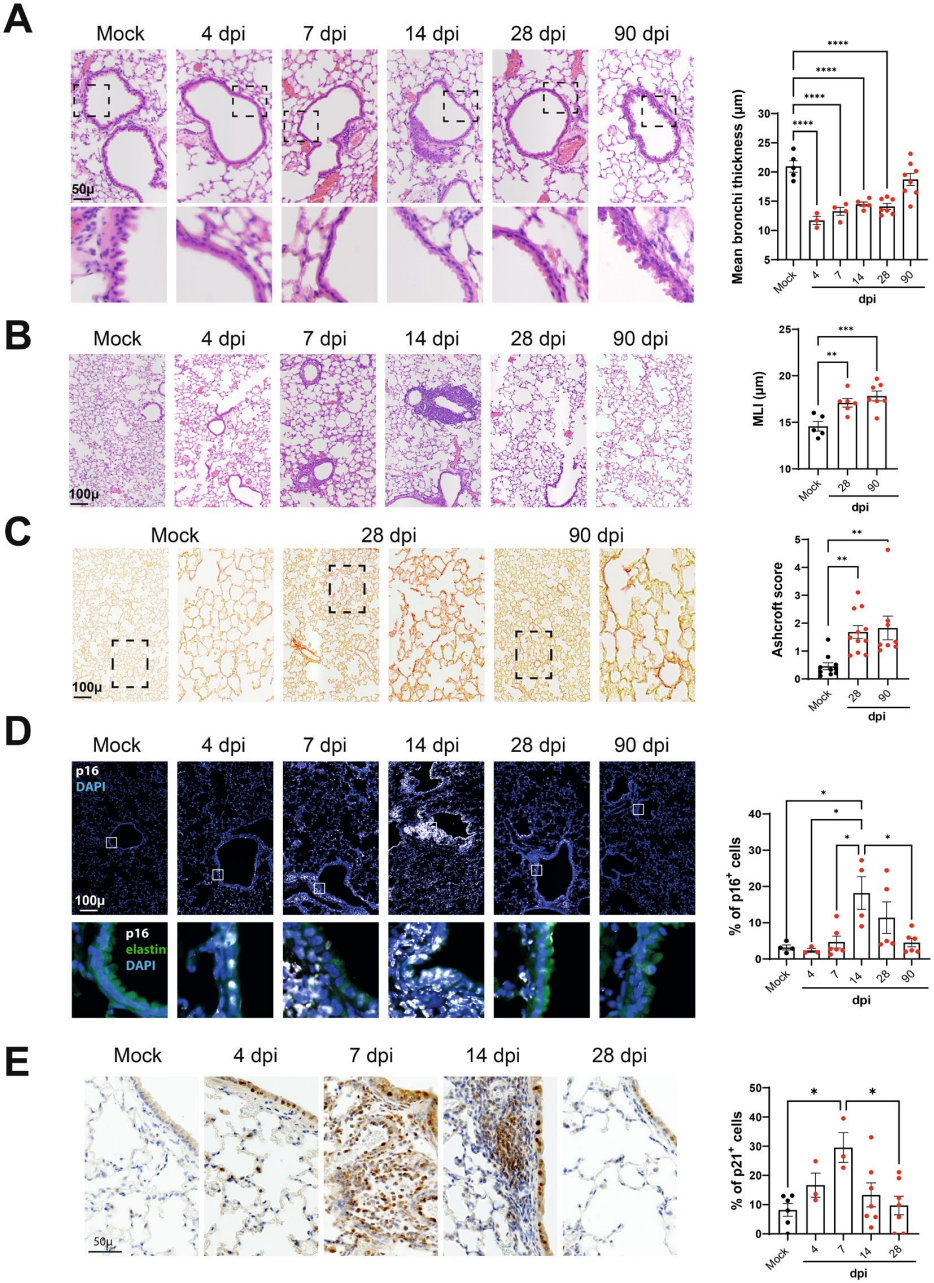
Time course of pulmonary pathological manifestations and p16 and p21 expression following IAV infection. (A) Left panels. Representative micrographs of H&E‐stained lung sections showing bronchi (upper panel) and bronchial wall (lower panel) of mock‐infected mice and IAV‐infected mice. The zoomed areas are indicated by rectangles. Right panel. Scatter‐plot graph showing bronchial wall thickness. (B) Left panels, Representative micrographs showing lung parenchyma. Right panel. Scatter plot showing mean liner intercept (MLI) measurements. (C) Left panels, Representative micrographs showing lung parenchyma stained with Sirius Red used to visualize collagen deposition (hallmark of lung fibrosis). Zoomed area are indicated by squares. Right panel, Scatter‐plot graph showing parenchymal fibrosis quantification according to Aschcroft score. (D) Left panel. Representative micrographs showing immunofluorescence of p16 (white) in lung cells. Blue—DAPI nuclear staining, green—elastin autofluorescence. The zoomed areas (lower panels) are indicated. (E) Left panel. Representative micrographs showing p21 expression by immunohistochemistry. (D, E) Right panel, Scatter‐plot graphs representing the percentage of p16‐positive (D) and p21‐positive (E) cells in the different groups of mice. (A–E) Scales are indicated (bar = 50 or 100 μm). Graphs represent individual values per mice and the mean ± SEM (*n* = 3–11). Significant differences were determined using a one‐way ANOVA followed by Bonferroni post hoc test (*p < 0.05, **p < 0.01, ***p < 0.001, ****p < 0.0001).

To assess the extent of IAV‐induced lung damage and the fate of senescent lung cells over time, we investigated mice exposed to a sublethal dose of H1N1p2009 until 90 days post‐infection (dpi) (for the viral load in lungs and body weight loss and recovery, see Figure S1A,B). A histochemical analysis (H&E staining) of lung sections revealed pulmonary lesions at 4 dpi that predominated at 7 and 14 dpi, including structural bronchial and alveolar lesions, inflammatory infiltrates, and airway epithelial abrasion that appeared complete in some areas (Figure 1A). On 28 dpi, the lungs appeared to have recovered partly from these structural changes, with a reduction in inflammatory infiltrates but persistence of abrasion of the airway epithelium (Figure 1A, lower panels). Interestingly, lung emphysema lesions (assessed by measurement of the mean linear intercept, Figure 1B) were observed at 28 dpi, together with the appearance of fibrotic lesions (as assessed with the modified Ashcroft score, Figure 1C). On 90 dpi, the bronchial airway epithelium had recovered partly, although the persistence of lung emphysema and fibrotic lesions was consistent with the establishment of a chronic lung disease (Figure 1A–C).

**FIGURE 2:**
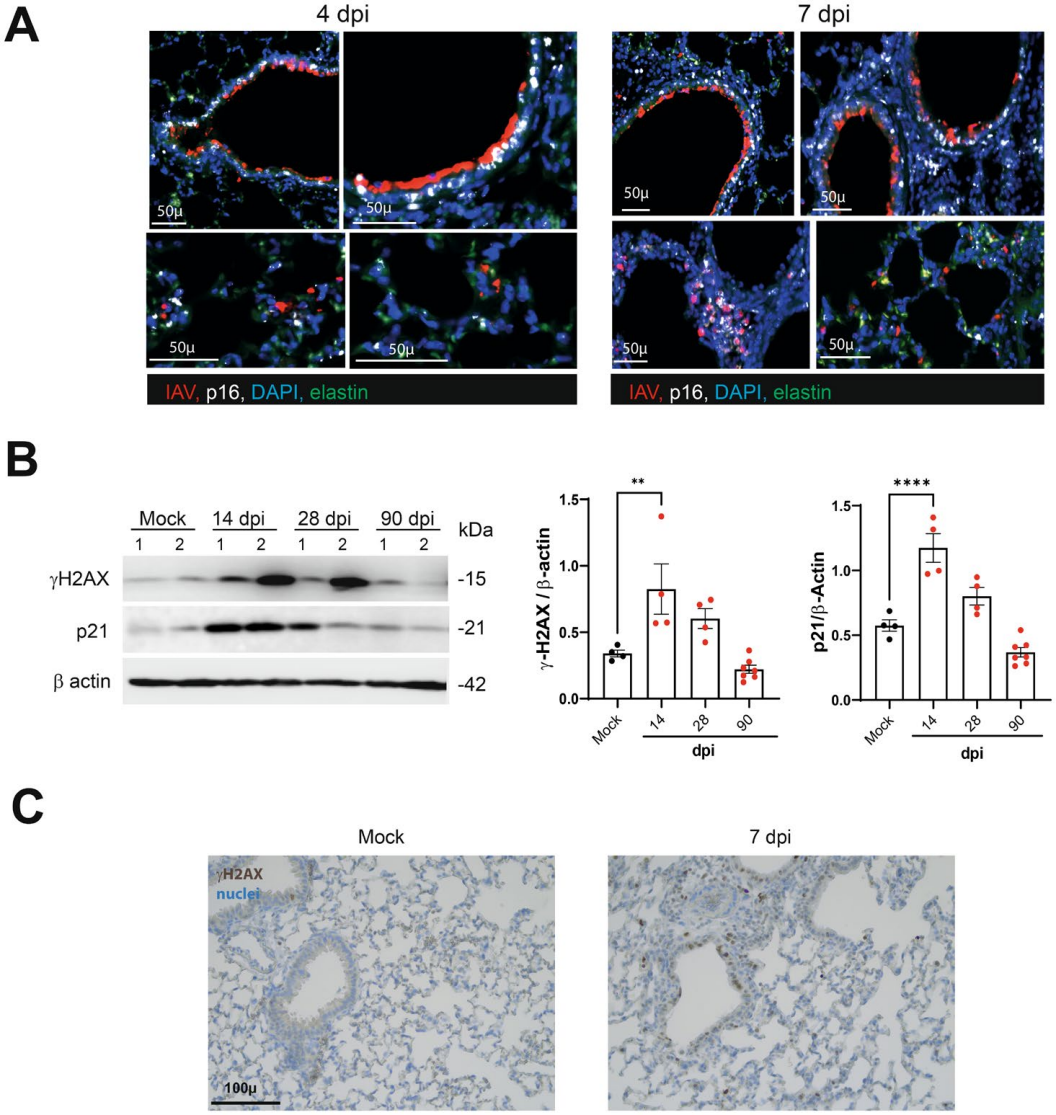
p16 and viral antigen and ©H2AX and p21 co‐expression in lungs during influenza. (A) Representative micrograph showing immunofluorescence of viral hemagglutinin (IAV, red) and p16 (white) in lung sections collected from IAV‐infected mice at 4 and 7 dpi. Bleu—DAPI nuclear staining, green, elastin autofluorescence). Scales are indicated. (B) Expression of gamma‐H2A.X and p21 protein in IAV‐infected whole lung homogenates as assessed by western blotting. The relative protein levels are normalized to β Actin. Graphs represent individual values per mice and the mean ± SD (*n* = 4–7). Significant differences were determined using a one‐way ANOVA followed by Bonferroni post hoc test (**p < 0.01, ****p < 0.0001). (C) Expression of gammaH2AX in the lungs from mock‐infected and IAV‐infected (7 dpi) mice by immunohistochemistry. Blue–Nuclear hematoxylin staining (bar = 100 μm).

Alongside these structural changes, immunolabeling analyses revealed a massive accumulation of cells expressing the senescence markers p16 and p21 (Figure 1D,E). Cells stained for p16 or p21 were detected as early as 4 dpi in the airway epithelium, with a peak for p21‐positive cells at 7 dpi and a peak for p16‐positive cells at 14 dpi. Lung cells stained for p16 or p21 were rarer but still present at 28 dpi, indicating that senescent lung cells persisted after the virus had been cleared. Consistent with these observations, thoracic bioluminescence was observed in p16luc/+ heterozygous mice at 7–14 dpi (Figure S1C). At 4 dpi, almost all p16‐positive cells were bronchial epithelial cells that also expressed viral antigen (Figure 2A). At 7 dpi, an extension of p16 staining was observed in the lung parenchyma in cells not expressing the viral antigen, indicating the spreading of the senescence process. Beyond 14 dpi, the expression of viral proteins became poorly detectable. At 90 dpi, overexpression of p16 and p21 was no longer observed (Figure 1D and not shown).

Elevated expression of the galactosidase beta 1 (GLB1), the enzyme mediating the senescence‐associated‐β‐galactosidase activity, is observed during cellular senescence (Lee et al. 2006; Sun et al. 2022). Expression of GLB1 was enhanced in IAV‐infected lungs as assessed by Western blotting (7 dpi, Figure S1D). Histone H2A.X phosphorylated at serine 139 (gamma‐H2A.X) is a marker of the DNA damage response associated with cellular senescence. As shown in Figure 2B, elevated levels of gamma‐H2A.X (and p21) were observed in the lungs of IAV‐infected mice with a peak at 14 dpi. Immunolabeling confirmed the enhanced expression of γ‐H2A.X in lung tissue during infection (Figure 2C). In contrast, the lung levels of the DNA damage marker 53BP1 decreased during IAV infection (Figure S1E). Such opposing changes in lung gamma‐H2A.X and 53BP1 expression have been recently reported in response to a severe acute respiratory syndrome coronavirus 2 (SARS‐CoV‐2) infection (Gioia et al. 2023), supporting a common response elicited by IAV and SARS‐CoV‐2. Taken together, in agreement with other studies (Kulkarni et al. 2019; Lv et al. 2022; Schulz et al. 2023), IAV infection leads to cellular senescence in the lungs and this senescence associates with lasting damage to lung tissue.

### 2.2 Delayed Recovery of the Bronchial Epithelium Is Related to the Persistence of Senescent Cells in the Peribronchial Area

**FIGURE 3:**
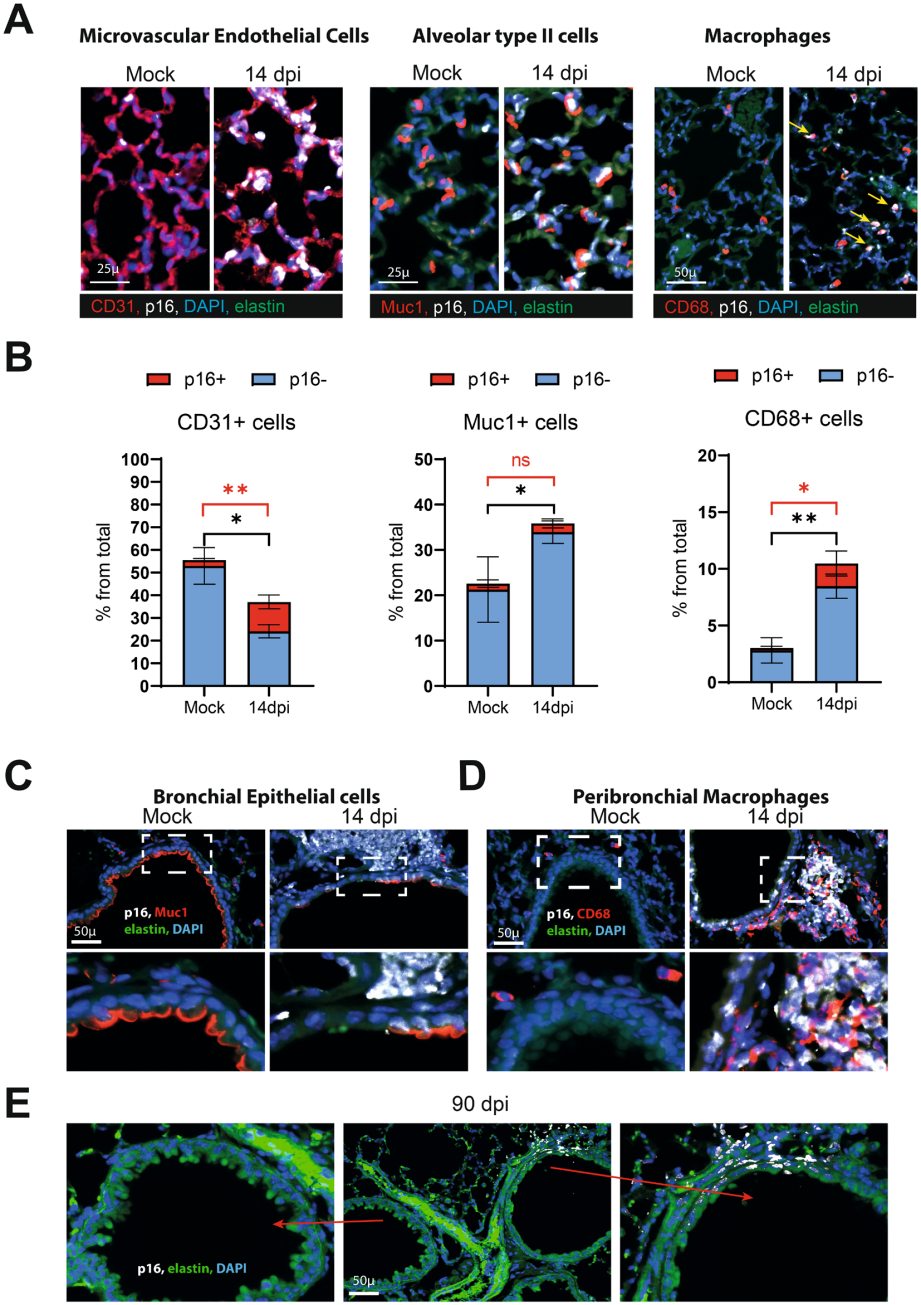
Identification of pulmonary cell types becoming senescent on 14 and 90 dpi. Representative micrographs showing immunofluorescence of p16 (white) in pulmonary cells co‐stained with cell‐type specific markers. Blue–DAPI nuclear staining, green–elastin autofluorescence (14 dpi). (A) Representative micrographs showing immunofluorescence of p16 (white) in microvascular endothelial cells (CD31), alveolar type II cells (Muc1) and macrophages (CD68). (B) The percentage of p16‐positive cells in each cell population is depicted (*n* = 3). Significant differences were determined using the two‐tailed Mann–Whitney *U* test (*p < 0.05, **p < 0.01). Of note, the total number of macrophages and parenchymal Muc1 stained cells were increased at 14 dpi, contrasting with a decreased number of lung endothelial cells (black asterisk). (C, D) Images showing p16 expression in peribronchial areas. Lung cells co‐stained either with (C) Muc1 (red, a marker of bronchial epithelial cells) or with (D) CD68 (red, a marker of macrophages). The zoomed areas (lower panels) are indicated by rectangles. (E) Micrographs showing immunofluorescence of p16 (white) in peribronchial area associated with the zone of defective epithelial layer (arrow, right images). Left image show normal bronchi on 90 dpi.

To further identify the lung cell types that undergo senescence upon IAV infection, we performed co‐immunolabeling for p16 and specific cell markers on 14 dpi (the peak of p16 staining). Pulmonary vascular and microvascular endothelial cells, bronchial and alveolar epithelial cells, and macrophages expressed p16 protein, as shown by double‐immunofluorescence staining for p16 and CD31, Muc1, and CD68, respectively (Figure 3). A significant proportion of endothelial cells (the frequency of which being decreased on 14 dpi) and macrophages (increased frequency) expressed p16 (Figure 3A,B). Given the presence of bronchial epithelium abrasion on 14 dpi, only a few of the remaining bronchial epithelial cells were co‐stained for p16 and Muc1 at this time point (Figure 3C). In fact, most of the p16‐positive cells in the surrounding bronchial walls were positive for CD68 (Figure 3D). At 90 dpi, most of the bronchi had a normal epithelium (Figure 3E). The bronchi that did not recover contained senescent cells in the vessel wall. Figure 3E shows samples from the same mouse lung: the absence of p16‐positive cells and the complete airway epithelium repair in one bronchus (left panel) contrasts with the persistence of p16‐positive cells and only partial repair in a nearby bronchus (right panel).

To determine whether these phenomena occurred in non‐human primates, we examined the lungs of macaques infected with IAV and sacrificed on 28 dpi (Figure S2). Immunohistochemical studies of lung sections revealed a large number of p16‐positive cells primarily at sites of persistent alveolar damage and in thrombotic pulmonary blood vessels, as we have described previously following a SARS‐CoV‐2 infection (Lipskaia et al. 2022). As we observed in mice, the airway epithelium had not recovered at 28 dpi, and this time point was associated with the persistence of p16‐positive cells in the bronchial wall (Figure S2).

The epithelial cell is IAV's first target. To assess whether IAV could directly induce epithelial lung cell senescence, we analyzed publicly available datasets from human bronchial epithelial cells (GSE71766) (Kim et al. 2015) and mouse epithelial alveolar cells sorted from IAV‐infected mice (GSE57008) (Stegemann‐Koniszewski et al. 2016) on 3 dpi. Strikingly, IAV infection resulted in an enrichment in cell senescence‐related genes and aging‐related genes in both human and mouse lung epithelial cells (Figure S3).

### 2.3 The Genetic Elimination of p16‐Expressing Cells Protects Against the Chronic Lung Damage Induced by Influenza

**FIGURE 4:**
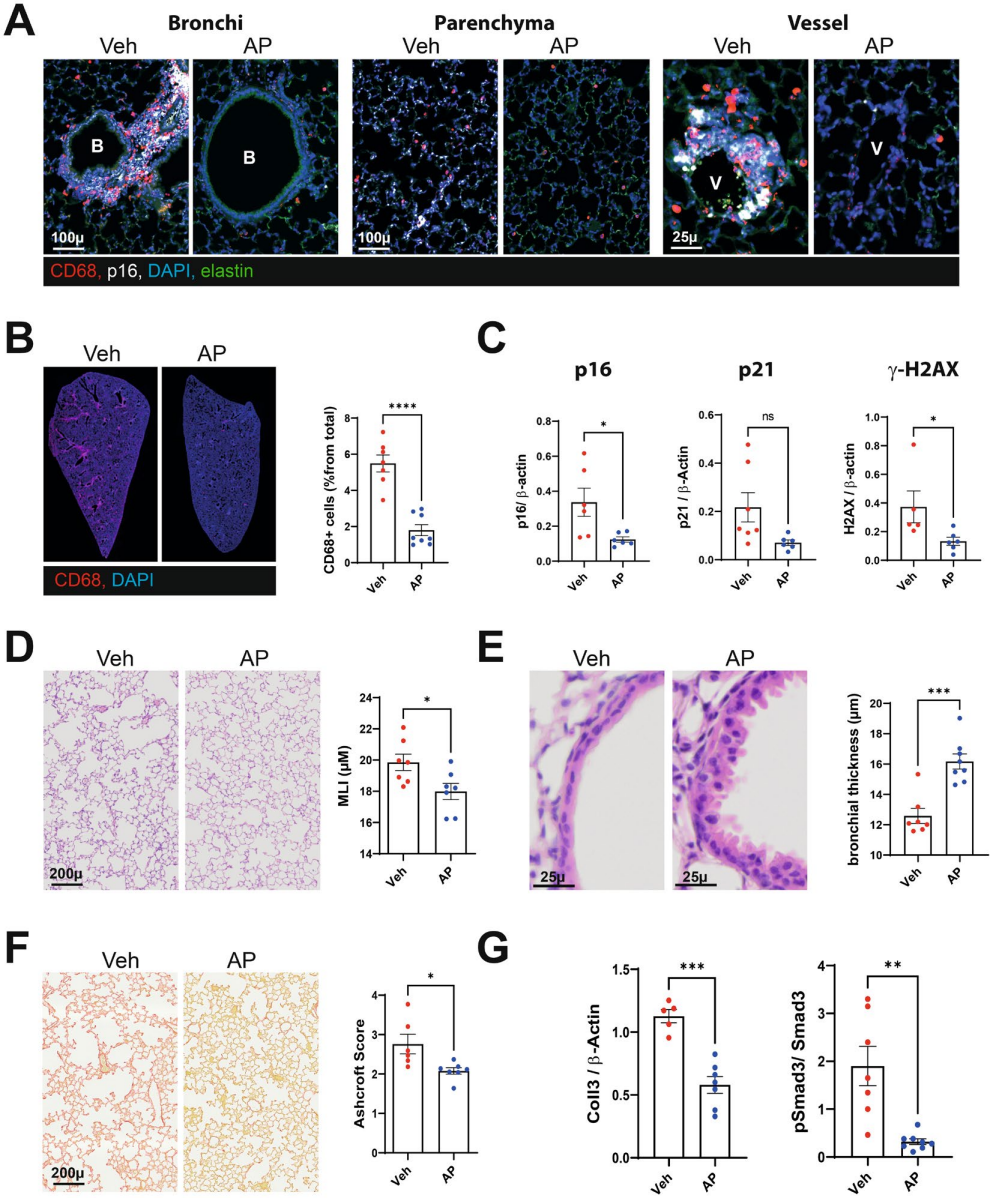
Consequences of genetic elimination of senescent cells on lung sequelae post‐influenza. p16‐ATTAC mice were intraperitoneally inoculated with AP20187 (0.5 mg/kg, twice weekly) starting the day before infection. Lungs from vehicle‐treated and AP20187‐treated mice were collected on 28 dpi. (A) Representative micrographs showing immunofluorescence of p16 (white) and CD68 (red) in lung cells. Blue–DAPI nuclear staining, green–Elastin autofluorescence. Bar—100 or 25 μm. (B) Effect of AP20187 treatment on macrophages infiltration in lung. Left panel, Representative whole lung scan showing CD68‐immunofluorescence (red). Blue–Dapi nucler staining. Right panel: Scatter plot showing the abundance of CD68 positive cells (% from total). Graphs represent individual values per mice and the mean ± SEM (*n* = 6–8). (C) Expression of p16, p21 protein and gammaH2AX in IAV‐infected whole lung homogenates as assessed by western blotting. The relative protein levels are normalized to β‐Actin (*n* = 5–8). (D) Effect of AP20187 treatment on lung emphysema. Left panel: Representative hematoxylin/eosin sections of lung from different group of mice. Bar = 200 μ. Right panel. Scatter plot showing mean liner intercept (MLI). (E) Effect of AP treatment on bronchial regeneration. Left panel: Representative micrographs showing bronchial wall. Hematoxylin/eosin staining. Bar 25 μ. Right panel. Scatter‐plot graph showing bronchial wall thickness. (F) Effect of AP20187 treatment on pulmonary fibrosis. Left panel: Representative Sirus‐Red stained sections of lung from different group of mice. Bar = 200 μ. Right panel. Scatter plot showing Ashcroft score. (G) Relative expression of the pulmonary fibrosis markers Coll3 and pSmad3 in whole lung homogenates as assessed by western blotting. The protein level of Coll3 was normalized to beta Actin and thatt of pSmad3 was normalized to Smad3. (B–G) Graphs represent individual values per mice and the mean ± SEM (*n* = 7–8). One of two representative experiments is shown. Significant differences was determined using the Mann–Whitney *U* test (*p < 0.05, **p < 0.01, ***p < 0.001, ****p < 0.001).

We next assessed the effects of senescent cell elimination on post‐influenza chronic lung damage. To this end, we studied p16‐ATTAC mice expressing an inducible suicide gene (caspase 8) under the control of the p16 promoter and activated by the rapalog AP20187 (Born et al. 2023). It is noteworthy that IAV‐associated lung damage was similar in p16‐ATTAC mice and wild type mice, with the presence of lung emphysema and lung fibrosis on 28 and 90 dpi (Figure S4A,B). Compared with vehicle, the initiation of AP20187 treatment at the time of the IAV infection effectively eliminated p16‐positive cells in the lung parenchyma and the airways (Figure 4A). In particular, the CD68‐positive cells that accumulated in perivascular and peribronchial areas were eliminated upon AP20187 treatment (28 dpi, Figure 4A,B). Accordingly, recurrent administration of AP20187 was associated with markedly lower lung p16, p21, and gamma‐H2A.X protein levels on 28 dpi (Figure 4C, Figure S4C). Although not significant, treatment with AP20187 ameliorated the time course of weight loss and recovery (Figure S4D). Lung emphysema was less intense in p16‐ATTAC mice treated with AP20187 than in vehicle‐treated counterparts (Figure 4D). Remarkably, the airway epithelium completely recovered in AP20187‐treated mice by 28 dpi; this indicated marked acceleration of the epithelial repair process (Figure 4E). Lastly, pulmonary fibrosis was reduced in AP20187‐treated p16‐ATTAC mice (Figure 4F). In line with these histological findings, treatment with AP20187 was associated with lower expression of collagen 1 alpha 1, collagen 3, and phospho‐SMAD proteins—the main markers of pulmonary fibrosis (Figure 4G, Figure S4E). Hence, the genetic removal of p16‐expressing cells is associated with amelioration of chronic post‐influenza lung damage.

### 2.4 The Removal of p16‐Expressing Cells Does Not Alter the Early Lung Response to IAV Infection

**FIGURE 5:**
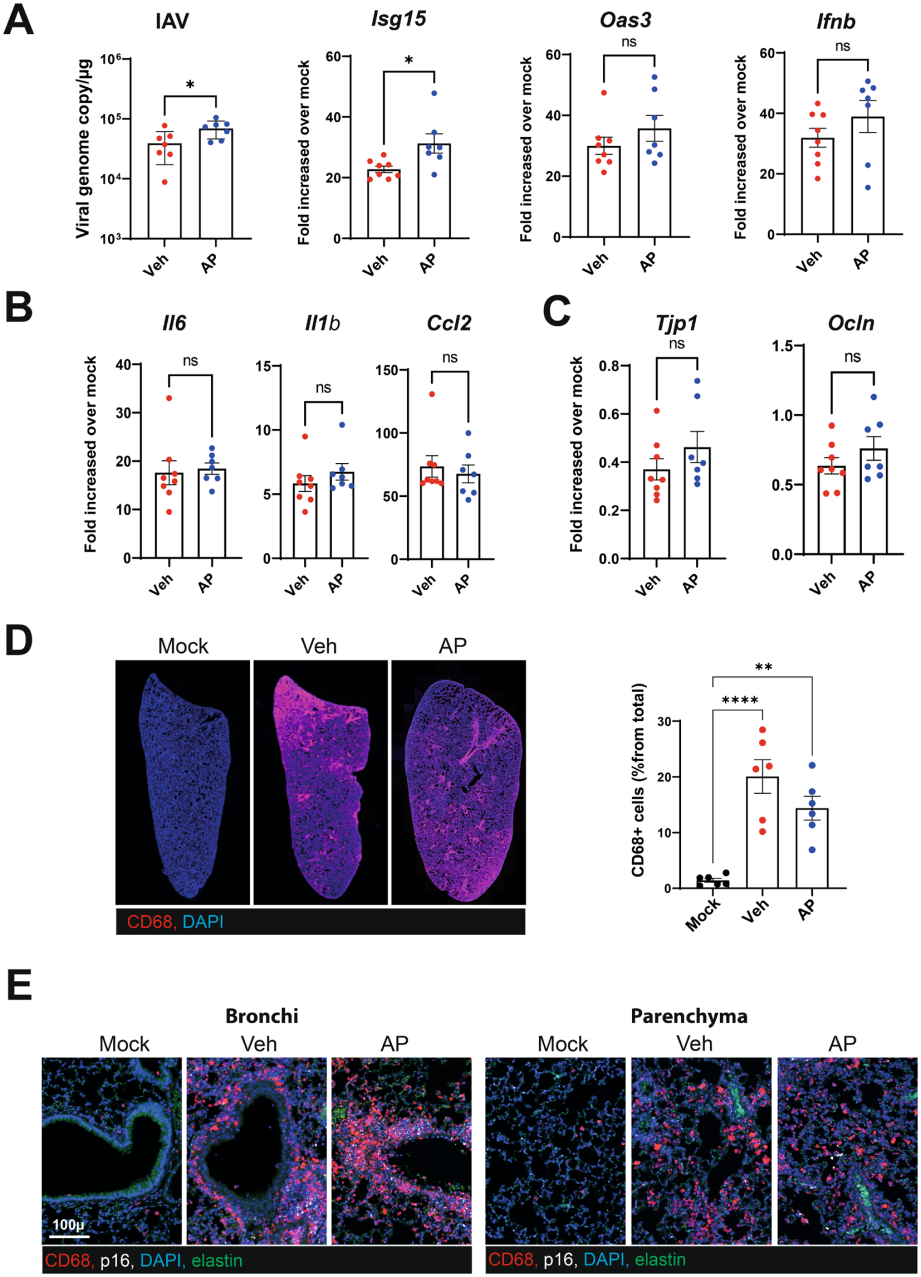
Consequences of senescent cells removal on the acute phase response of influenza. Lungs from vehicle‐treated and AP20187‐treated mice were collected on 7 dpi. (A) Viral load (left panel) and the interferon‐stimulated genes *Isg15*, *Oas3* and *Ifnb* were quantified by quantitative RT‐qPCR. (B, C) mRNA copy numbers (for inflammatory genes in panel B and for genes related to barrier functions in panel C) were quantified by RT‐qPCR. (A–C) The data are expressed as the mean ± SD fold change relative to average gene expression in mock‐infected animals. (D Left panel, Representative micrographs showing CD68‐labeled lungs from mock‐infected and IAV‐infected (vehicle and AP20187‐treated) p16‐ATTAC mice (magnification: ×2.5). Left panel, The percentage of CD68‐postive cells is indicated for each group. (E) Lungs were co‐labeled with anti‐CD68 and anti‐p16 antibodies. (A–D) Graphs represent individual values per mice and the mean ± SD (*n* = 6–8). One of two representative experiments is shown. Significant differences were determined using the two‐tailed Mann–Whitney *U* test (A–C) or using a one‐way ANOVA followed by Bonferroni post hoc test (D) (*p < 0.05, **p < 0.01, ****p < 0.0001).

The ablation of virus‐induced senescent cells has been shown to decrease the pulmonary viral load and/or inflammatory marker expression soon after a SARS‐CoV‐2 infection (Lee et al. 2021; Tsuji et al. 2022; Aguado et al. 2023). To investigate whether the protection against the above‐described long‐term effects of IAV was due to an effect during the early phases of infection, AP20187‐treated p16‐ATTAC mice were analyzed on 7 dpi (the peak of the inflammatory response). Compared with vehicle‐treated mice, AP20187‐treated mice had a slightly but significantly higher pulmonary load of IAV (Figure 5A, left panel, in a quantitative RT‐PCR). Expression of *Isg15* (but not *Ifnb* nor *Oas3*) was significantly elevated upon treatment with AP20187 (Figure 5A, right panels). Furthermore, the depletion of p16‐expressing cells did not change the pulmonary expression of transcripts encoding inflammatory markers like interleukin (IL)‐6, IL‐1β, and CCL2 (Figure 5B). It is well known that influenza disrupts the epithelial barrier (Short et al. 2016). The IAV‐induced reduction in the expression of epithelial cell markers (such as zonula occludens‐1 (encoded by *Tjp1*) and the tight junction protein occludin (encoded by *Ocln*) was not modified by AP20187 treatment (Figure 5C)). Inflammatory monocytes play a key role in pulmonary pathogenesis during influenza (Schmit et al. 2022). As expected, the recruitment of inflammatory monocytes/macrophages to the lungs was intense on 7 dpi—particularly in the perivascular and peribronchial areas (Figure 5D,E). Most of these cells were p16‐negative at this time point. Accordingly, AP20187 treatment failed to significantly lower the number of inflammatory monocytes/macrophages (Figure 5D,E). We conclude that the removal of p16‐expressing cells does not affect the early, acute response to influenza.

### 2.5 Pharmacological Elimination of Senescent Cells Favors Airway Epithelial Repair Post‐Influenza

**FIGURE 6:**
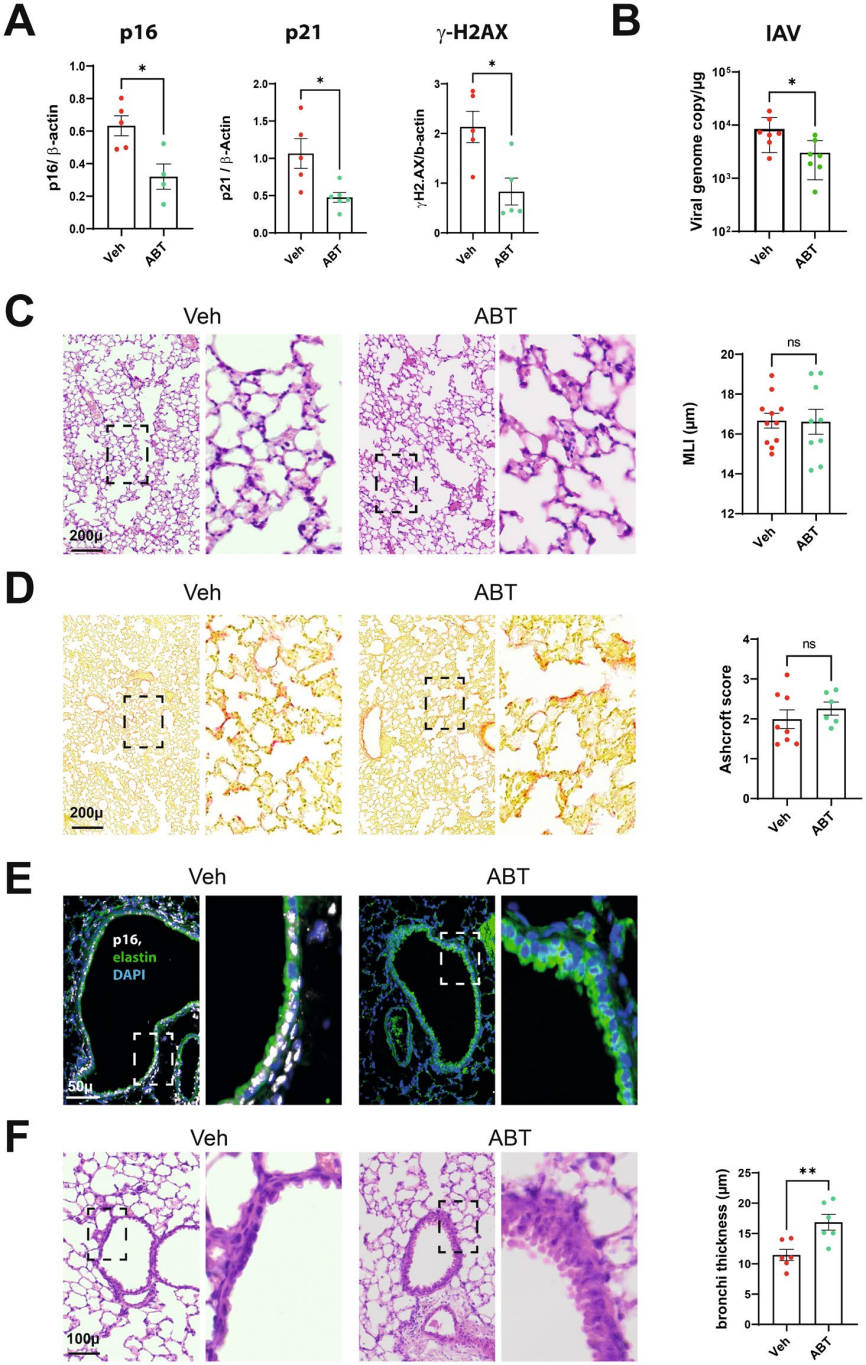
Consequences of ABT‐263 treatment on lung sequelae post‐influenza. (A) Expression of p16, p21 protein and **♥**‐H2AX in IAV‐infected whole lung homogenates as assessed by western blotting (28 dpi). The relative protein levels are normalized to beta‐Actin (*n* = 5–6). (B) Viral load as assessed by quantitative RT‐PCR (7 dpi). (C, D) Left panel. Lung sections (28 dpi) were stained with hematoxylin/eosin (C) or Sirius red (D) (bar = 200 μ). Right panel. Scatter plot showing mean liner intercept (MLI) and parenchymal fibrosis quantification. (E, F) Effect of senescent cells elimination on bronchial regeneration (bar = 100 μ) (28 dpi). (E) Representative micrographs showing immunofluorescence of p16 (white) in lung cells of vehicle‐treated and ABT‐263‐treated mice. Blue–DAPI nuclear staining. The zoomed areas are indicated by rectangles. Bar—100 μm. (F) Left panel, Representative micrographs of H&E‐stained lung sections. Right panel, Scatter‐plot graph showing bronchial wall thickness. (A–F) Graphs represent individual values per mice and the mean ± SD (*n* = 4–8). Significant differences was determined using the Mann–Whitney *U* test (*p < 0.05, **p < 0.01).

The use of senolytic drugs is a potentially feasible clinical approach for reducing the harmful effects of senescent cells (Justice et al. 2019; Chaib et al. 2022). To determine whether the pharmacological elimination of senescent cells had much the same effects as genetic senolysis in p16‐ATTAC mice, wild type mice were treated with ABT‐263 (navitoclax). The latter drug induces senescent cell apoptosis by counteracting the anti‐apoptotic function of B cell lymphoma‐2 (Bcl‐2) family members (Zhu et al. 2016). The oral administration of ABT‐263 was initiated 24 h after the start of the IAV infection and continued until the end of the experiment (28 dpi). As assessed by Western blotting, ABT‐263 efficiently reverted the virus‐induced expression of the senescence markers p16, p21, and gamma‐H2A.X in lung extracts (Figure 6A, Figure S5A). In line with these data, immunofluorescence staining revealed a reduction (but not total abrogation) in the number of p16‐ and p21‐positive cells in the lung parenchyma and bronchi (Figure S5B,C). ABT‐263 treatment did not alter body weight loss and recovery (Figure S5D), slightly reduced the viral load (Figure 6B) but had no significant effects on the expression of transcripts encoding ISGs, inflammatory genes, and epithelial barrier markers (7 dpi, Figure S5E and not shown). Quantification of IL‐6, IL‐1β, TNF‐α and IFN‐gamma proteins in lung extracts confirmed the lack of anti‐inflammatory effect of ABT‐263 (Figure S5F).

We then analyzed the potential effect of senolysis on long‐term pulmonary sequelae. In contrast to the p16‐ATTAC system, recurrent ABT‐263 treatment did not prevent the development of lung emphysema or lung fibrosis on 28 dpi (Figure 6C,D). We then investigated whether ABT‐263 inoculation impacts the airway epithelium repair. Interestingly, ABT‐263 treatment led to complete recovery of the airway epithelium on 28 dpi, together with the elimination of p16‐positive cells in the airway walls (Figure 6E,F). We conclude that the pharmacological depletion of senescent cells by means of ABT‐263 favors airway epithelial repair during influenza.

### 2.6 Elimination of p16‐Positive Cells During the Recovery Phase of Influenza Ameliorates Bronchial Epithelium Recovery

**FIGURE 7:**
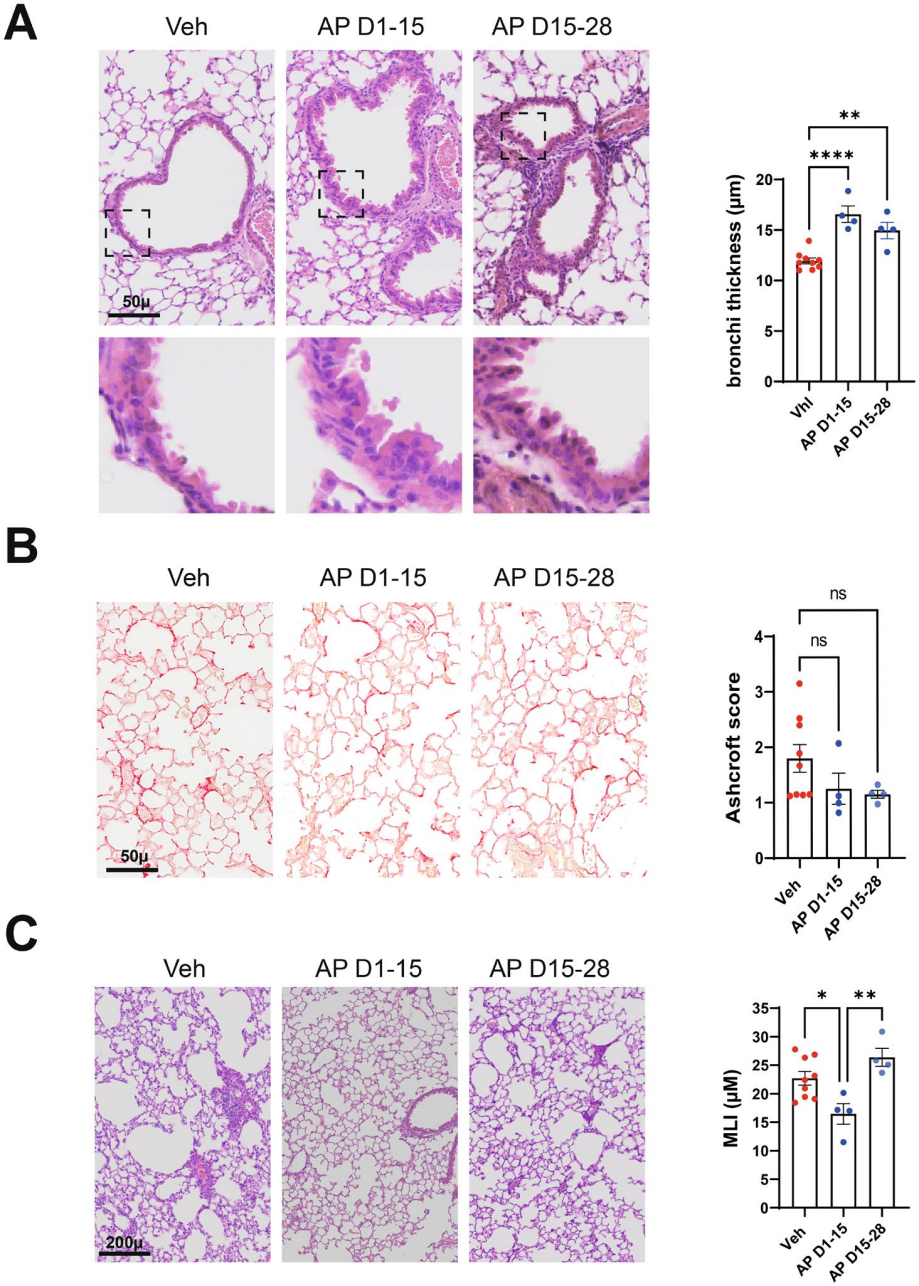
Consequences of senescent cells elimination during the early and recovery phases of influenza on lung sequelae post‐influenza. p16‐ATTAC mice were treated with AP20187 from 1 to 15 dpi (D1‐15) or from 15 to 28 dpi (D15‐28). Lungs from vehicle‐treated and AP20187‐treated mice were collected on 28 dpi. (A) Left panel, Effect of AP treatment on bronchial regeneration. Representative micrographs showing bronchial wall (hematoxylin/eosin staining, bar = 50 μ). (B) Left panel, Effect of AP treatment on pulmonary fibrosis. Representative Sirius‐Red stained sections of lung from different group of mice. Bar = 50 μ. (C) Left panel, Effect of AP20187 treatment on lung emphysema (hematoxylin/eosin staining, bar = 200 μ). (A–C) Graphs represent individual values per mice and the mean ± SEM (*n* = 4–9). Significant differences were determined using a one‐way ANOVA followed by Bonferroni post hoc test (*p < 0.05, **p < 0.01, ****p < 0.001).

A question of clinical importance is whether early or late removal of senescent cells can improve pulmonary sequelae post‐influenza. As senescent (p16‐positive) cells persist after the clearance of the virus, we investigated the possibility that their genetic removal at late time points could have an impact on lung sequelae. To this end, p16‐ATTAC mice were treated with AP20187 from 15 dpi to the sacrifice at 28 dpi (AP D1‐15). Another group of mice was treated at the time of the IAV infection to 15 dpi (AP D15‐28). Remarkably, both protocols led to a complete restoration of the bronchial epithelium (Figure 7A) and to a trend towards improvement of pulmonary fibrosis (Figure 7B). Protection against emphysema was only observed when treatment was initiated at the time of infection but not on 15 dpi (Figure 7C).

## 3 Discussion

Acute viral pneumonia leads to prolonged pulmonary damage as a result of excessive inflammation and/or defective repair processes (Herold et al. 2015; Narasimhan et al. 2024). The mechanisms underlying these defective repair processes remain to be fully determined. Our findings substantially expand the understanding of how respiratory viruses induce long‐term sequelae in lungs. In our preclinical model of influenza, lung emphysema and fibrotic lesions developed on 28 dpi and then persisted until 90 dpi. In parallel, the airways completely lacked epithelium on 28 dpi, and some sites remained denuded at 90 dpi. Concomitantly with these structural changes, senescent cells accumulated in lungs. In line with other reports (Schulz et al. 2023; Lv et al. 2022; Kulkarni et al. 2019), p16‐ and p21‐positive cells appeared at early time points. This occurred with the increase in gamma‐H2A.X, consistent with a DNA damage response considered a primary mechanism of virus‐induced lung cell senescence and known to be induced by IAV (Li et al. 2015). At 14 dpi, p16‐positive cells, mostly endothelial cells and alveolar type II cells—both of which are involved in the structural maintenance of the lung—were distributed throughout the lung parenchyma. In parallel, senescent macrophages were present in the peribronchial and perivascular areas. Cellular senescence persisted after 28 dpi (when the virus was no longer detected) suggesting a paracrine effect of SASP on senescence (Schulz et al. 2023). The coincidence between the accumulation of senescent cells and the onset of the lung repair process suggested that the two phenomena are functionally connected. Interestingly, the bronchial walls at the denuded sites stained positive for p16, which suggests that persistent p16‐expressing cells have a negative effect on the epithelial repair process. Consistent with these observations, mice in which senescent cells had been eliminated (using a suicide gene strategy in p16‐ATTAC mice or treatment with the senolytic drug ABT263 in wild‐type mice) showed almost complete recovery of the airway epithelium on 28 dpi. This observation indicates that the clearance of senescent cells accelerated the airway epithelium repair process. This is of major importance, given the role of airway epithelial cells as a barrier to secondary bacterial or fungal infections—a major cause of death in patients admitted for acute respiratory failure (McCullers 2014). Moreover, delayed repair of the bronchial epithelium may favor the induction of chronic bronchitis, with persistent coughing and a potential exacerbation of asthma (particularly in children). It is well known that macrophages can alter repair processes (Su et al. 2021; Pervizaj‐Oruqaj et al. 2024). In our system, p16‐positive peribronchial macrophages were depleted by AP20187; this suggests that senescent macrophages might have a role in limiting the physiological re‐epithelization process. The mechanisms underlying this potential negative influence of senescent macrophages remain to be identified.

Previous studies have shown that the elimination of senescent cells can protect against lung emphysema or pulmonary fibrosis in relevant (non‐infectious) animal models (Lehmann et al. 2017; Schafer et al. 2017; Kaur et al. 2023). In the p16‐ATTAC mice genetic model, the elimination of p16‐expressing cells prevented the development of both pulmonary emphysema and fibrosis post‐influenza. This dual effect is suggestive of a common disease mechanism. Several mechanisms might contribute to reducing these long‐term sequelae in our setting. One might involve the attenuated effect of senescent cell clearance on the early (acute) phase of IAV infection. In our setting, and in line with a previous report (Kakkola et al. 2013), the genetic or pharmacological depletion of senescent cells did not strongly affect the viral load (with opposite effect of AP20187 and ABT‐263). Furthermore, the depletion of senescent cells failed to reduce local inflammation and epithelial damage early after infection. These data suggest that the beneficial role of senescent cell depletion in our setting is not due to inhibition of detrimental inflammatory responses early after infection. Thus, the most likely mechanistic explanation of our present findings involves a dampening effect of senescent cells on lung repair processes. In line, elimination of senescent cells after the acute phase period (from 15 dpi to the sacrifice) ameliorated epithelium structures post‐influenza. As discussed above, senescent macrophages are likely to be important in this setting. Idiopathic pulmonary fibrosis is thought to result from inadequate repair of sustained alveolar epithelial injury by alveolar type II cells. Furthermore, pulmonary emphysema can be induced by the senescence of alveolar type II cells or endothelial cells (Sarker et al. 2015; Houssaini et al. 2018; Hu et al. 2024). Thus, impairment of the repair function of senescent alveolar type II cells and endothelial cells might account for the pulmonary emphysema and fibrosis observed in IAV‐infected mice. An additional mechanism potentially involved in fibrosis and emphysema might involve senescent macrophages. Macrophages can undergo cellular senescence during a SARS‐CoV‐2 infection (Lee et al. 2021) and a recent study reported a significant similarity between COVID‐19‐associated macrophages and profibrotic macrophage populations identified in idiopathic pulmonary fibrosis (Wendisch et al. 2021). IL‐1β production by (senescent) macrophages might play a role in driving pulmonary fibrotic sequelae post‐influenza (Narasimhan et al. 2024).

Our results in p16‐ATTAC mice differed somewhat from those obtained after ABT‐263 treatment. Indeed, the latter effectively enhanced the bronchial epithelium's recovery but did not reduce lung emphysema or fibrosis. ABT‐263 targets cells overexpressing the anti‐apoptotic marker Bcl‐2. The harmful effects of apoptosis of Bcl‐2‐expressing cells and other unexpected effects might mask the potentially beneficial effect of senolysis in our experimental setting. Indeed, it was recently shown that ABT‐263 favors the development of fibrotic lesions and/or long‐term SASP production in some situations (Victorelli et al. 2023). The development of more targeted senolytics is essential to control pulmonary sequelae post‐influenza.

To the best of our knowledge, the present study is the first to have shown that the depletion of senescent cells in young individuals counters the long‐term sequelae of influenza. This might also be true for infections with other respiratory viruses. The finding that IAV infection can cause long‐term effects via the persistence of senescent cells is of major clinical importance for both adults and children; IAV infection is common in young infants whose lungs are still maturing. Indeed, it now appears that lung emphysema or chronic obstructive pulmonary disease in many adult patients is the end result of lung function impairments established in young adulthood and that are not greatly accentuated thereafter (Lange et al. 2015). The negative effect of senescent cells on the lung repair process might contribute significantly to these impairments in early adulthood. The present study had some limitations. The mouse data should be interpreted with caution. Although post‐acute sequelae clearly persist, this preclinical model does not replicate all the features of influenza in humans. However, our observations in macaques are consistent with those in mice and thus are more likely to have clinical relevance. It remains to be seen whether the harmful effect of senescent cells on long‐term lung disorders during influenza translates into worse pulmonary function. Another limitation of the present study relates to our focus on ABT‐263—a drug that targets Bcl‐2 family proteins. The specific targeting of senescent cells should now be studied using other classes of senolytics. It would be interesting to investigate the senotherapeutic potential of dasatinib (a tyrosine kinase inhibitor) and quercetin (a kinase‐inhibiting flavonoid) shown to alleviate cellular senescence‐associated (lung) diseases in humans (Hickson et al. 2019; Justice et al. 2019). It is noteworthy that the combination of dasatinib and quercetin reduced acute‐phase lung disease in preclinical models of COVID‐19 (Lee et al. 2021; Pastor‐Fernández et al. 2023). The long‐term effect of this treatment remains to be determined. In conclusion, our findings suggest that cellular senescence is a potential new target for controlling the long‐term consequences of influenza virus and potentially other viruses known to induce lung disease post‐infection. One striking observation was the accelerated recovery of the airway epithelium following the elimination of senescent cells, the role of which is crucial for patient outcomes and the occurrence of pulmonary complications during severe influenza. Therapeutic applications could be expected from these data.

## 4 Experimental Procedures

### 4.1 Animal and Ethics

Specific pathogen‐free C57BL/6J mice (8‐10‐week‐old, male) were purchased from Janvier (Le Genest‐St‐Isle, France). Mice expressing luciferase under the control of the p16 promoter (heterozygous p16LUC/+) mice were obtained from Dr. N. Sharpless (University of North Carolina School of Medicine, Chapel Hill, NC) (Burd et al. 2013). Mice expressing a killer gene construct driven by the p16 promoter (p16‐ATTAC mice) have been described (Born et al. 2023). Mice were maintained in a biosafety level 2 facility at the Animal Resource Center at the Institut Pasteur de Lille for at least 1 week prior to use to allow appropriate acclimatization. Mice were fed standard rodent chow (SAFE A04) (SAFE, Augy, France) and water *ad libitum*. Experiments in mice complied with current national and institutional regulations and ethical guidelines (Institut Pasteur de Lille/B59‐350009). Protocols were approved by the regional Animal Experimentation Ethics Committee (Comité d'Ethique en Expérimentation Animale, Hauts de France, CEEA 75) and the French Ministry of Higher Education and Research (Ministère de l'Education Nationale, de l'Enseignement Supérieur et de la Recherche) (authorization numbers: APAFIS#27417‐2020093019014739 v3). Cynomolgus macaques ( *Macaca fascicularis* ) originating from Mauritian AAALAC certified breeding centers were used in this study. All animals were housed in IDMIT infrastructure facilities (CEA, Fontenay‐aux‐roses), under BSL‐2 and BSL‐3 containment when necessary (Animal facility authorization #D92‐032‐02, Préfecture des Hauts de Seine, France) and in compliance with European Directive 2010/63/EU, the French regulations and the Standards for Human Care and Use of Laboratory Animals, of the Office for Laboratory Animal Welfare (OLAW, assurance number #A5826‐01, US). The protocols were approved by the institutional ethical committee “Comité d'Ethique en Expérimentation Animale du Commissariat à l'Energie Atomique et aux Energies Alternatives” (CEtEA #44) under statement number A18_025. The study was authorized by the “Research, Innovation and Education Ministry” under registration number APAFIS# 17836‐2018112716574241 v1.

### 4.2 Reagents

AP20187 (HY‐13992, Cliniscience, Nanterre, France) was dissolved in ethanol, then diluted in 10% polyethylene glycol 400% and 0.2% Tween 20. ABT‐263 (Cliniscience) was dissolved in dimethyl sulfoxide, then diluted in 10% PEG400 and 60% Phosal 50 PG.

### 4.3 IAV Infection, Bioluminescence Imaging, and Depletion of Senescent Cells

For infection, mice were anesthetized by intraperitoneal administration of 1.25 mg of ketamine and 0.25 mg of xylazine in 100 μL of phosphate buffered saline (PBS), and then intranasally (i.n.) infected with 50 μL of PBS containing 100 pfu of H1N1 A/California/04/2009 (pdm09) strain. Mice treated i.n. with PBS served as controls (non‐infected mice). The appearance of senescent lung cells was detected by bioluminescence tissue imaging using IAV‐infected heterozygous p16LUC/+ mice. Isoflurane‐anesthetized mice were injected intravenously with D‐luciferin substrate (L6882, Sigma, St Quentin Fallavier, France). The bioluminescence signal was acquired during 10 min with Photon Imager Optima (Biospace Lab, Paris, France). Results were analyzed using PhotoAcquisition software (Biospace Lab). To genetically eliminate senescent cells, p16‐ATTAC mice were treated intraperitoneally with AP20187 at 0.5 mg/kg three times a week starting the day before infection until the sacrifice. Mice were also treated between 1 and 15 dpi or between 15 and 28 dpi. To pharmacologically eliminate senescent (Bcl2‐expressing) cells, mice were administered in a daily manner by oral gavage with ABT‐263 (50 mg/kg) starting 1 day before infection until the sacrifice. Vehicle served as controls. Mice in both groups were sacrificed at different time points post‐infection. The right lung was quickly removed and immediately snap‐frozen in liquid nitrogen and then stored at −80°C until total RNA extraction for real‐time polymerase chain reaction analysis. The left lung was fixed by intratracheal infusion of 4% paraformaldehyde aqueous solution at a transpleural pressure of 30 cm H_2_O for histological or immunofluorescence studies.

Adults, female cynomolgus macaques (macaca fascicularis) aged 2–3 years were exposed to a total dose of 2.1 × 10^6^ pfu of H1N1/2009/Cal07 via the combination of intranasal, oropharyngeal, and intratracheal routes, using atropine (0.04 mg kg^−1^) for pre‐medication and ketamine (5 mg kg^−1^) with medetomidine (0.05 mg kg^−1^) for anesthesia. Animals were observed daily and clinical exams were performed at baseline and twice weekly on anesthetized animals using ketamine (5 mg kg^−1^) and medetomidine (0.05 mg kg^−1^). Body weight was recorded daily Animals were euthanized on 28 dpi. PBS‐treated macaques served as controls. Lung tissues were fixed in 4% PBS buffered formaldehyde for 2 days, rinsed in PBS, transferred to ethanol (70%) and then processed into paraffin‐embedded tissue blocks.

### 4.4 Transcriptomic Analysis

For transcriptomic analyses of datasets from IAV‐infected human bronchial epithelial cells and mouse lungs, data were extracted from the GEO dataset public database, and Gene Set Enrichment Analysis (GSEA) was performed using GSEA v2.0.13 software with default parameters (www.broadinstitute.org/gsea/). GSEA analyses were done on the datasets corresponding to human bronchial epithelial cells (BEAS‐2B) (GSE71766) and sorted type II alveolar epithelial cells after mouse infection (GSE57008), 3 days after IAV infection in both cases.

### 4.5 Immunofluorescence

Paraffin‐embedded sections of lung were deparaffinized using xylene and a graded series of ethanol di‐lutions then processed for epitope retrieval using Antigen retrieval buffer (100X Tris‐EDTA buffer, pH 9.0, ab93684, Abcam) according to the manufacturer's instructions. For nuclear immunolabeling, tissues were permeabilized with 0.1% Triton X‐100 in PBS for 10 min. Saturation was achieved using Dako antibody diluents with 10% goat serum. For immunolabeling with primary antibody produced in mouse we used the M.O.M. (mouse on mouse) immunodetection kit, basic (ref. BMK‐2202, Vector Lab) according to manufacturer instructions. For double staining, first and second primary antibodies were diluted in Dako antibody diluents with 3% goat serum then incubated for 1 h at 37°C in a humidified chamber. After PBS washes, the sections were covered with secondary antibody (Dako antibody diluents with 3% of goat serum mixed with mouse or rabbit Alexa Fluor 555 or Alexa Fluor 660 [Thermofisher]) for 40 min at 37°C in a humidified chamber. The sections were secured with fluorescent mounting medium containing DAPI and protected with coverslips. Double‐immunolabeling for p16 and H1N1 HA was performed by using Tyramide SuperBoost Kits with Alexa Fluor Tyramides (B400923 and B40916, Invitrogen) according to manufacturer instructions. Fluorescence was recorded using an Axio Imager M2 imaging microscope (Zeiss, Oberkochen, Germany) and analyzed on digital photographs using Image J software (imagej.nih.gov/ij/). The following primary antibodies were used for immunofluorescence: anti‐CDKN2A/p16INK4a (1:200 for common immunolabeling and 1:1000 for labelling using tyramide, ab54210, Abcam); anti‐CD31 (PECAM [platelet endothelial cell adhesion molecule]; 1:1000, ab182981, Abcam); anti‐CD68 (1:100, ab 283,654, Abcam); anti‐MUC1(1:200, ab109185, Abcam); anti‐H1N1 HA (1:1000, PA5‐34929, Invitrogen). To quantify p16, CD31, Muc1 and CD68‐positive cells, the entire tissue sections were scanned using Axioscan Z1 (Zeiss, Germany) and then subjected to digital image analysis using QuPath open software for bioimage analysis (https://qupath.github.io). Cells were identified and counted by DAPI nuclear staining. Cells were classified as either p16 positive or negative based on the average intensity of nuclear Alexa 630 staining, then classified as either CD31, Muc1, CD68 positive or negative based on the average intensity of cytosolic Alexa 555 staining. Double‐labeled cells were identified by nuclear 630 and cytosolic 555 average intensity.

### 4.6 Immunohistochemistry

Briefly, paraffin‐embedded lung sections were deparaffinized using the PT Module (Epredia) and Dewax and HIER Buffer H, pH 9.0 (TA‐999‐DHBH, Epredia) according to the manufacturer's instructions. Immunohistochemical labelling was performed on the Autostainer 360‐2D (Epredia). After PBS‐Tween 20 buffer (PBT010, SkyTek Laboratories) washed, slides were treated with Peroxide Block solution (ACA500, SkyTek Laboratories). The nuclei were then permeabilized with 0.1% Triton X‐100 in PBS for 10 min. SkyTek Laboratories' EZ Block solution (EZB125) was used to reach saturation. For immunolabeling, sections were incubated with the primary antibody diluted in Normal Antibody Diluent (ABB 500, EZB 125, SkyTek Laboratories). The following primary antibodies were used: rabbit anti‐p21 polyclonal antibody (28248‐1‐AP, Proteintech, 1:200), rabbit anti‐53BP1 polyclonal antibody (NB100‐304, Novusbio, 1:200) and rabbit monoclonal anti‐phospho‐Histone H2A.X antibody (20E3, #9718, 1:500, Cell signaling Technology). The sections were then washed with PBST buffer and incubated with the anti‐rabbit HRP‐labeled polymer (K4003, Dako). The sections were then washed, incubated with DAB working solution (ImmPACT DAB EqV, Peroxidase substrate, SK‐4103, Vector Laboratories), and rinsed twice with distilled water. The nuclei were stained using hematoxylin (HAQ500, SkyTek Laboratories). The sections were then rinsed twice with water and a bluing solution (0.1%) of sodium bicarbonate. The slides were then dehydrated, cleared, secured with a permanent, non‐aqueous medium, and covered with coverslips for protection. To quantify p21‐ and 53BP1‐positive cells, the entire tissue sections were scanned using Axioscan Z1 (Zeiss, Germany) and then subjected to digital image analysis using QuPath open software for bioimage analysis (https://qupath.github.io). Cells were classified as either positive or negative based on the average intensity of nuclear DAB staining.

### 4.7 Histological Analysis

For morphometry studies, 5 μm‐thick sagittal sections along the greatest axis of the left lung were cut in a systematic manner. Lung emphysema was measured using mean linear intercept methods on hematoxylin–eosin (H&E) coloration. Briefly, 20 fields/animal light microscope fields at an overall magnification of 500 were overlapped with a 42‐point, 21‐line eyepiece according to a systematic sampling method from a random starting point. MLI measurement was performed by an independent operator using semi‐automated measurement of MLI. Both methods (manual and semi‐automated) performed by two independent observers provided the same results. To measure bronchial epithelial thickness, the entire tissue sections were scanned using Axioscan Z1 (Zeiss, Germany) and then epithelial thickness was measured using ZEN 3.0 software (ZEISS, Rueil Malmaisson, France) for bioimage analysis. The samples were examined microscopically and morphometrically by two investigators who were unaware of the origin of the material. For each animal, at least 10 transversally cut terminal bronchioles with an adequate cross‐sectional profile (less than 10% of variation in maximal and minimal diameter) were measured, and only airways with a visible full perimeter were analyzed. The average epithelium thickness was determined by measuring the distance between the basal membrane limit and the apical membrane limit in at least 10 regions of bronchia. Values measured for each of the 10 airways were averaged to provide a single data point for each animal. Lung fibrosis was quantified on lung sections stained with Sirius Red (Picro Sirius Red Stain Kit, ab150681, Abcam, Cambridge, UK) using the modified Ashcroft scale. Briefly, the lungs were scanned microscopically with a 20‐fold objective, which allowed the evaluation of fine structures while also providing a sufficiently broad view. The entire section was examined by inspecting each field in a raster pattern. Areas with dominating tracheal or bronchial tissue were omitted. The grades were summarized and divided by the number of fields to obtain a fibrotic index for the lung.

### 4.8 Western Blotting

Lung extracts were prepared as described (Delval et al. 2023). Proteins were then separated using 12 or 15% SDS‐PAGE and then transferred from the gel to a PVDF membrane. The antibodies used are as follows: anti‐β‐actin (A1978, Sigma Aldrich); anti‐γ‐H2AX (Ser139; #9718, Cell Signaling Technology); anti‐CDKN2A/p16INK4a (#PA5‐20379, Thermo Fisher Scientific); anti‐CDKN1A/p21 (#ab 8224, Abcam); anti‐collagen I (Col1A1) (#ab260043, Abcam); anti‐collagen III (#ab184993, Abcam); anti‐phospho‐Smad3 (Ser423/425) (#9520, Cell Signaling); and anti‐Smad3 (#9523, Cell Signaling). The detection was made by using the appropriate horseradish peroxidase‐conjugated secondary antibody (Sigma Merck). The signal was detected using an enhanced chemiluminescence detection system (GE Healthcare). The unsaturated images were acquired using a ChemiDoc MP imaging system (Bio‐Rad), and the signal densities were quantified using Image Studio Lite ver 5.2 (LI‐COR). To normalize, an antibody directed against β actin (#4970, Cell Signaling) was used.

### 4.9 Quantification of Viral Load and Determination of Gene Expression by Quantitative RT‐PCR

Viral load in the lungs was determined by quantifying viral RNA encoding the M1 protein (segment 7) (Heumel et al. 2024). RNA was reverse‐transcribed with SuperScript II Reverse Transcriptase (Invitrogen, Waltham, MA) using primers specific for M1 (5′‐TCTAACCGAGGTCGAAACGTA‐3′). Quantitative PCR was performed using TaqMan Universal PCR Master Mix (Applied Biosystems, Waltham, MA), using primers for M1 (Table 1) and M1‐specific TaqMan probe (FAM) 5′‐TTTGTGTTCACGCTCACCGTGCC‐3′ (TAMRA). Amplification was performed using the QuantStudio 5 K Flex Real‐Time PCR System (Applied Biosystems). Assessment of gene expression was performed by quantitative RT‐PCR using standard procedures. RNA was reverse‐transcribed using a High‐Capacity cDNA Archive Kit (Life Technologies, Carlsbad, CA, USA). The resulting cDNA was amplified using SYBR Green‐based real‐time PCR and the QuantStudio 5 K Flex Real‐Time PCR System (Applied Biosystems). The primers used are listed in Table 1. Relative mRNA levels were determined according to the 2^−ΔΔ^Ct (cycle thresholds) method by comparing (i) the PCR Ct for the gene of interest and the housekeeping gene (ΔCt) and (ii) the ΔCt values for the treated and control groups (ΔΔCt). Data were normalized against the expression of the *Gapdh g*ene and expressed as fold‐change over the mean gene expression level in mock‐treated mice.

**TABLE 1:** Sequences of oligonucleotides used in this study.

*Gapdh*	Forward 5′‐GCAAAGTGGAGATTGTTGCCA‐3′	*Oas3*	Forward 5′‐GTGGCACCGATGTCGAACTC‐3′
	Reverse 5′‐GCCTTGACTGTGCCGTTGA‐3′		Reverse 5’‐AGCAACATTCGCATGGCA‐3′
*Ccl2*	Forward 5′‐GCAGCAGGTGTCCCAAAGAA‐3′	*Ifnb*	Forward 5′‐TGGGTGGAATGAGACTATTGTTG‐3′
	Reverse 5′‐TCATTTGGTTCCGATCCAGGT‐3′		Reverse 5′‐CTCCCACGTCAATCTTTCCTC‐3′
*Il1b*	Forward 5′‐TCGTGCTGTCGGACCCATA‐3′	*Ocln*	Forward 5′‐AGCAGCCCTCAGGTGACTGTTATT‐3′
	Reverse 5′‐GTCGTTGCTTGGTTCTCCTTGT‐3′		Reverse 5′‐ACGACGTTAACTCCTGAACAAGCA‐3′
*Il6*	Forward 5′‐CAACCACGGCCTTCCCTACT‐3′	*Tpj1*	Forward 5′‐AGGTCTTCGCAGCTCCAAGAGAAA‐3′
	Reverse 5′‐CCACGATTTCCCAGAGAACATG‐3′		Reverse 5′‐ATCTGGCTCCTCTCTTGCCAACTT‐3′
*Isg15*	Reverse 5′‐GGCCACAGCAACATCTATGAGG‐3′	*IAV*	Forward 5′‐AAGAACAATCCTGTCACCTCTGA‐3′
	Reverse 5′‐CTCGAAGCTCAGCCAGAACTG‐3′		Reverse 5′‐CAAAGCGTCTACGCTGCAGTCC‐3′

### 4.10 Quantification of Cytokines in Lungs

Lung extracts were lysed in cold RIPA buffer (50 mM Tris–HCl pH 7.4, 150 mM NaCl, 1 mM EDTA, 1% Triton X‐100), 0.1% sodium deoxycholate supplemented with 1 mM PMSF and protease inhibitors (Roche Diagnostics, Basel, Switzerland), and centrifuged at 10000*g* for 5 min at 4°C to remove debris. The protein concentrations of clarified lysates were quantified using the PierceTM BCA protein assay kit (ThermoFisher Scientific). Samples were normalized to a single protein concentration (1 mg/mL) using the same lysis buffer. Cytokines were quantified as the Olink^R^ target 48 mouse cytokine kit exactly as described by the manufacturer (Olink Proteomics AB, Upssala, Sweden).

### 4.11 Statistical Analysis

Statistical analyses were performed using GraphPad Prism 9 software (San Diego, CA). One‐way or two‐way ANOVA followed by Bonferroni post hoc test were used to compare the means of more than two independent groups. Comparison between two groups was performed by using the Mann–Whitney *U* test. Unless otherwise stated, data are expressed as individual values and mean ± SEM or ±SD.

## Author Contributions

Serge Adnot and François Trottein conceived and supervised the study. Larissa Lipskaia, Lou Delval, Valentin Sencio, Serge Adnot, and François Trottein designed the experiments, and Lou Delval, Valentin Sencio, and Fabiola Silva Angulo performed the animal experiments. Larissa Lipskaia, Lou Delval, and Vincent Gros performed immunohistochemistry and immunofluorescence. Larissa Lipskaia, Amal Houssaini, Emmanuelle Born, Elisabeth Marcos, Shariq Abid, and Mira Goekyildirim managed the lung preparations, interpretation of lung structure, and assessment of pulmonary biological parameters. Amal Houssaini performed the western blotting; Lou Delval and Séverine Heumel performed the RT‐PCR. Jean Michel Flaman and David Bernard analyzed the transcriptomic data. Vanessa Contreras and Roger Le Grand performed the experiments in macaques. Larissa Lipskaia, Lou Delval, Valentin Sencio, David Bernard, François Trottein, and Serge Adnot analyzed the resulting data. Larissa Lipskaia designed the figures, and François Trottein and Serge Adnot drafted the manuscript. All the authors revised the manuscript and provided critical comments. François Trottein and Serge Adnot obtained funding.

## Conflicts of Interest

The authors declare no conflicts of interest.

## Supporting information


**Figure S1.** Induction of lung cell senescence following IAV infection in mice. Mice were intranasally (i.n.) infected with 50 μL of PBS containing (or not, in a mock sample) 100 p.f.u. of H1N1 A/California/04/2009 (pdm09). (A) Infected mice were sacrificed at different time points and viral load was measured in the whole lungs by quantitative RT‐PCR. Graphs represent individual values per mice and the mean ± SD (*n* = 5–6). The dashed line indicates the limit of detection. On 28 dpi, no viral transcript was detected (not shown). Significant differences were determined using a one‐way ANOVA followed by Bonferroni post hoc test. (B) Body weight loss and body weight regain during the course of infection (mean ± SD) (*n* = 8/group). (C) Thoracic bioluminescence of IAV‐infected p16luc/+ heterozygous mice. (D) Expression of Glb1 protein in IAV‐infected whole lung homogenates as assessed by western blotting. Representative western blottings are shown. The relative protein levels are normalized to β actin (mean ± SEM, *n* = 4–7). (E) Representative micrographs showing 53BP1 expression by immunohistochemistry. Right panels, Scatter‐plot graphs representing the percentage of p53BP1‐positive cells (mean ± SEM, *n* = 3–9). Significant differences were determined using a one‐way ANOVA followed by Bonferroni post hoc test (*p < 0.05, **p < 0.01, ***p < 0.001, ****p < 0.0001).

**Figure S2.** Pathological manifestations in the lungs of cynomolgus macaques following an IAV infection. (A) Histological (H&E staining) analysis (mock and 28 dpi). (B) Identification of senescent cells by immunofluorescence with p16 (red) (mock and 28 dpi). Blue—DAPI nuclear staining, green—elastin autofluorescence. Scales are indicated. Senescent cells were identified in the area of fibrosis and alveolar enlargement (upper panel, A and B), in the zone of bronchial epithelium destruction (middle panel A and B), and around damaged pulmonary vessels (lower panel, A and B). EpC—bronchial epithelial cells; EC—vascular endothelial cells.

**Figure S3.** Enrichment in expression of genes involved in cellular senescence and aging in human bronchial epithelial cells and mouse lungs infected by IAV. Data were extracted from public database and GSEA were performed using the GSEA v2.0.13 software using default parameters. All gene set files for this analysis were obtained from GSEA website (www.broadinstitute.org/gsea/). (A, B) Human bronchial epithelial cells (BEAS‐2B) were infected with IAV. Data were extracted from the public transcriptomic dataset GSE71766. GSEA analysis displayed expression enrichment in cellular senescence (A) and aging (B) signatures in influenza infected human bronchial epithelial cells 3 days post‐infection. (C, D) Mice were infected with IAV. Three days later, type II alveolar epithelial cells were collected and transcriptome analysis was performed. Data were extracted from public transcriptomic dataset GSE57008. GSEA analysis also displayed expression enrichment in cellular senescence (C) and aging (D) signatures in influenza infected lung mouse cells.

**Figure S4.** Analysis of lung emphysema and pulmonary fibrosis in IAV‐infected p16‐ATTAC mice. (A, B) Lungs were collected on 28 and 90 dpi. Lung sections were stained with hematoxylin/eosin (A) or Sirius red (B) (bar = 200 μ). Left panels, Representative micrographs. Right panels, scatter plot showing mean linear intercept (MLI) and parenchymal fibrosis quantification. (C) Representative western blotting showing the expression of p16, p21, γ‐H2A.X, and beta actin in vehicle and AP20187‐treated IAV‐infected mice (28 dpi) (whole lung homogenates). (D) Effect of AP20187 treatment on body weight loss and body weight regain during the course of infection (*n* = 8/group). (E) Left panel, representative western blotting showing the expression of collagen 1 alpha 1 (Coll1A1), collagen 3 (Coll3), and unphosphorylated and phosphorylated Smad3 (whole lung homogenates). Right panel, Scatter‐plot graphs representing the relative expression of (*n* = 7‐8/group). Significant differences were determined using a one‐way ANOVA followed by Bonferroni post hoc test (A, B) or the two‐tailed Mann–Whitney *U‐*test (E) (*p < 0.05, ***p < 0.001).

**Figure S5.** Effect of ABT‐263 on senescent cell marker expression and on influenza outcomes. (A) Western blotting showing the expression of p16, p21, and γH2AX and β‐actin in vehicle‐ and ABT‐263‐treated mice (28 dpi) (whole lung homogenates). (B, C) Elimination of p16‐positive cells (white) and p21‐positive cells (brown) by ABT‐263 as assessed by immunohistochemistry (28 and 7 dpi, respectively). Bar = 50 μ. (D) Effect of ABT‐263 treatment on the kinetics of body weight loss and body weight regain. (E) Expression of ISGs and inflammatory genes by quantitative RT‐PCR (7 dpi). (F) Quantification of inflammatory cytokines in lung extracts. (D–F) *n* = 7‐8/group.

## Data Availability

The data that support the findings of this study are available from the corresponding author upon reasonable request.
