# Supplementary figures and images for: Virus‐Induced Cellular Senescence Causes Pulmonary Sequelae Post‐Influenza Infection
Source: Aging Cell. 2025 Jun 20;24(9):e70140. doi: 10.1111/acel.70140 (PMC12419848; doi:10.1111/acel.70140)

Supplemental Figure 1

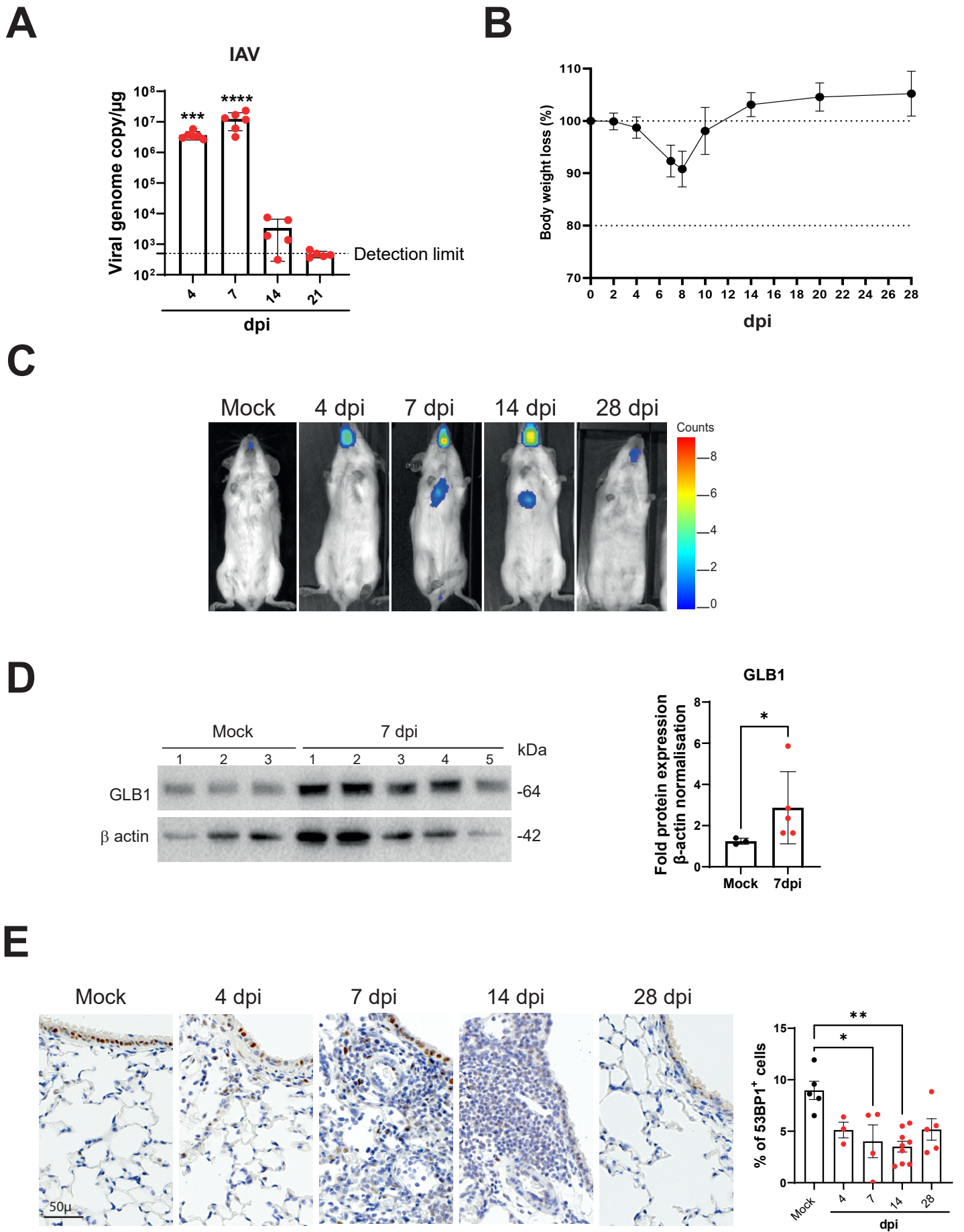

Supplement: Supplementary file 1 — Figure S1. Induction of lung cell senescence following IAV infection in mice. Mice were intranasally (i.n.) infected with 50 μL of PBS containing (or not, in a mock sample) 100 p.f.u. of H1N1 A/California/04/2009 (pdm09). (A) Infected mice were sacrificed at different time points and viral load was measured in the whole lungs by quantitative RT‐PCR. Graphs represent individual values per mice and the mean ± SD (n = 5–6). The dashed line indicates the limit of detection. On 28 dpi, no viral transcript was detected (not shown). Significant differences were determined using a one‐way ANOVA followed by Bonferroni post hoc test. (B) Body weight loss and body weight regain during the course of infection (mean ± SD) (n = 8/group). (C) Thoracic bioluminescence of IAV‐infected p16luc/+ heterozygous mice. (D) Expression of Glb1 protein in IAV‐infected whole lung homogenates as assessed by western blotting. Representative western blottings are shown. The relative protein levels are normalized to β actin (mean ± SEM, n = 4–7). (E) Representative micrographs showing 53BP1 expression by immunohistochemistry. Right panels, Scatter‐plot graphs representing the percentage of p53BP1‐positive cells (mean ± SEM, n = 3–9). Significant differences were determined using a one‐way ANOVA followed by Bonferroni post hoc test (*p < 0.05, **p < 0.01, ***p < 0.001, ****p < 0.0001). [file ACEL-24-e70140-s005.pdf]

Supplemental Figure 2

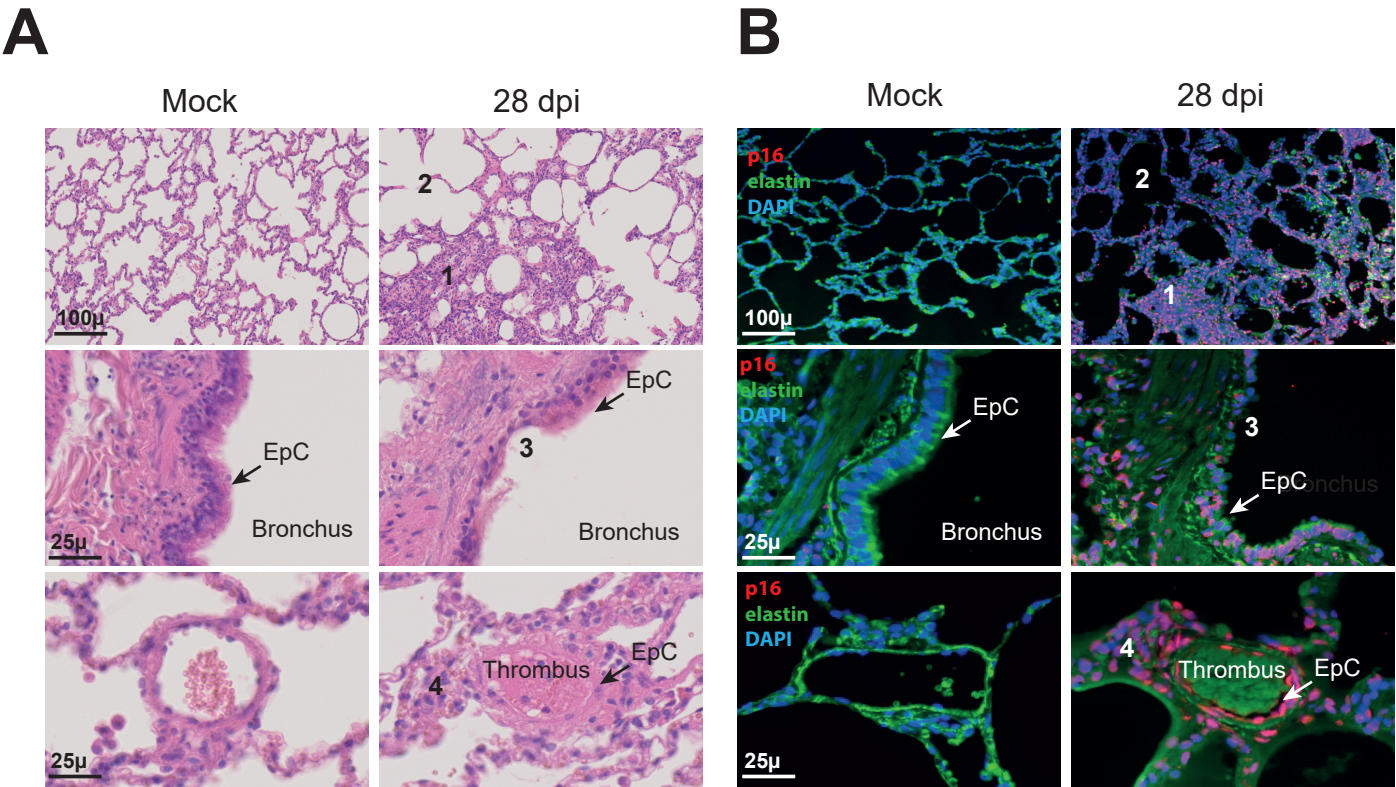

Supplement: Supplementary file 2 — Figure S2. Pathological manifestations in the lungs of cynomolgus macaques following an IAV infection. (A) Histological (H&E staining) analysis (mock and 28 dpi). (B) Identification of senescent cells by immunofluorescence with p16 (red) (mock and 28 dpi). Blue—DAPI nuclear staining, green—elastin autofluorescence. Scales are indicated. Senescent cells were identified in the area of fibrosis and alveolar enlargement (upper panel, A and B), in the zone of bronchial epithelium destruction (middle panel A and B), and around damaged pulmonary vessels (lower panel, A and B). EpC—bronchial epithelial cells; EC—vascular endothelial cells. [file ACEL-24-e70140-s001.pdf]

Supplemental Figure 3

A

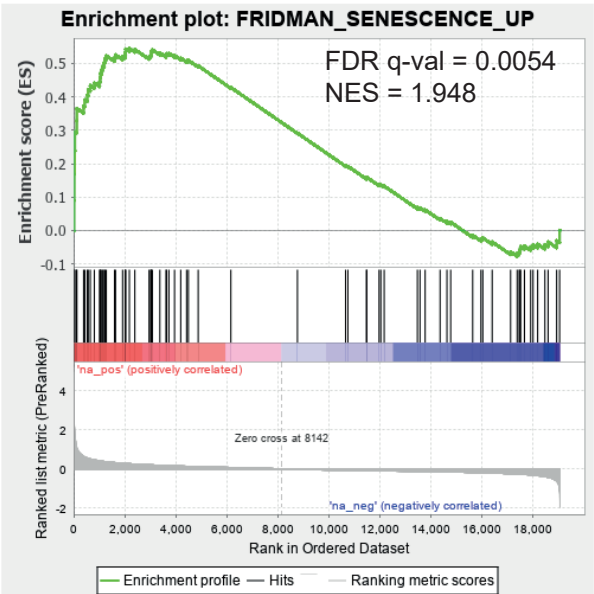

B

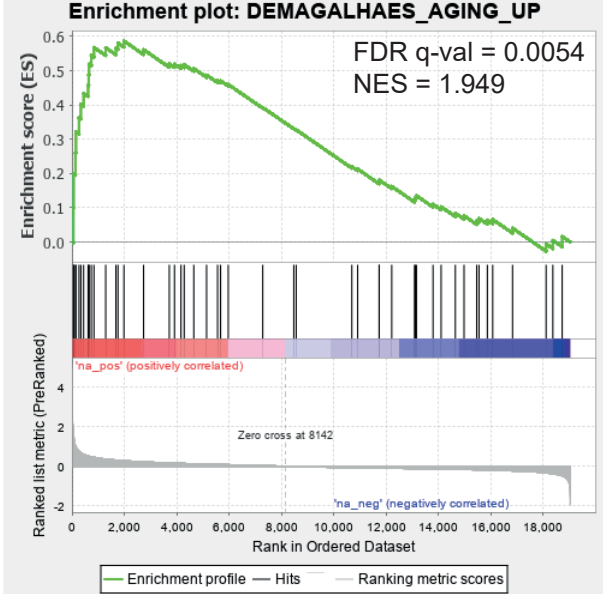

C

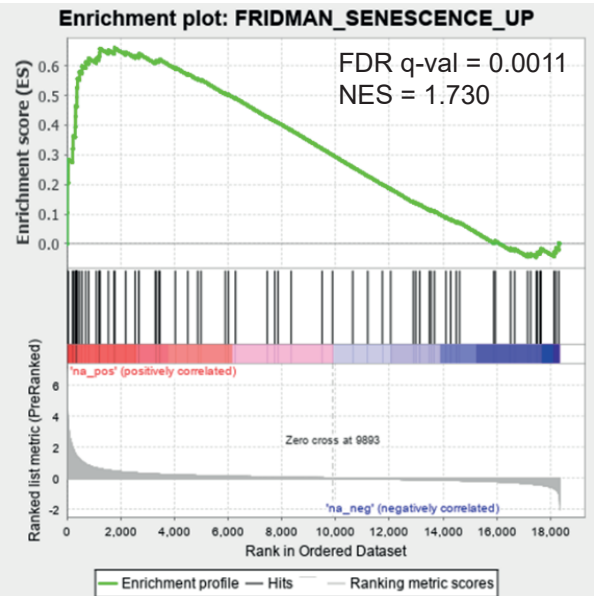

D

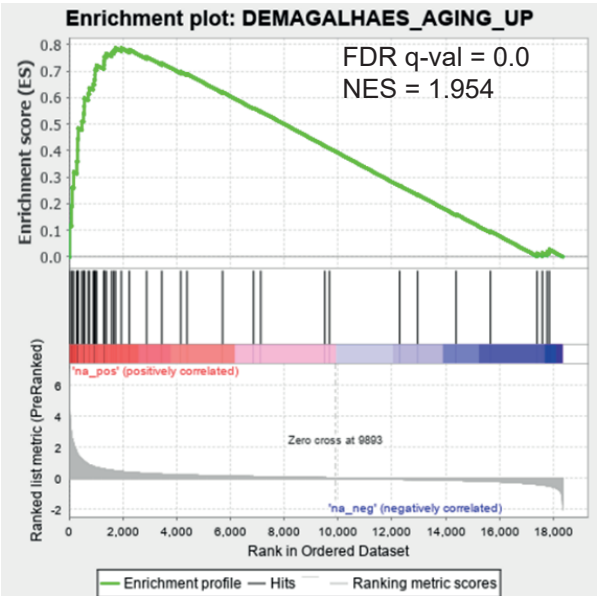

Supplement: Supplementary file 3 — Figure S3. Enrichment in expression of genes involved in cellular senescence and aging in human bronchial epithelial cells and mouse lungs infected by IAV. Data were extracted from public database and GSEA were performed using the GSEA v2.0.13 software using default parameters. All gene set files for this analysis were obtained from GSEA website (www.broadinstitute.org/gsea/). (A, B) Human bronchial epithelial cells (BEAS‐2B) were infected with IAV. Data were extracted from the public transcriptomic dataset GSE71766. GSEA analysis displayed expression enrichment in cellular senescence (A) and aging (B) signatures in influenza infected human bronchial epithelial cells 3 days post‐infection. (C, D) Mice were infected with IAV. Three days later, type II alveolar epithelial cells were collected and transcriptome analysis was performed. Data were extracted from public transcriptomic dataset GSE57008. GSEA analysis also displayed expression enrichment in cellular senescence (C) and aging (D) signatures in influenza infected lung mouse cells. [file ACEL-24-e70140-s003.pdf]

Supplemental Figure 4

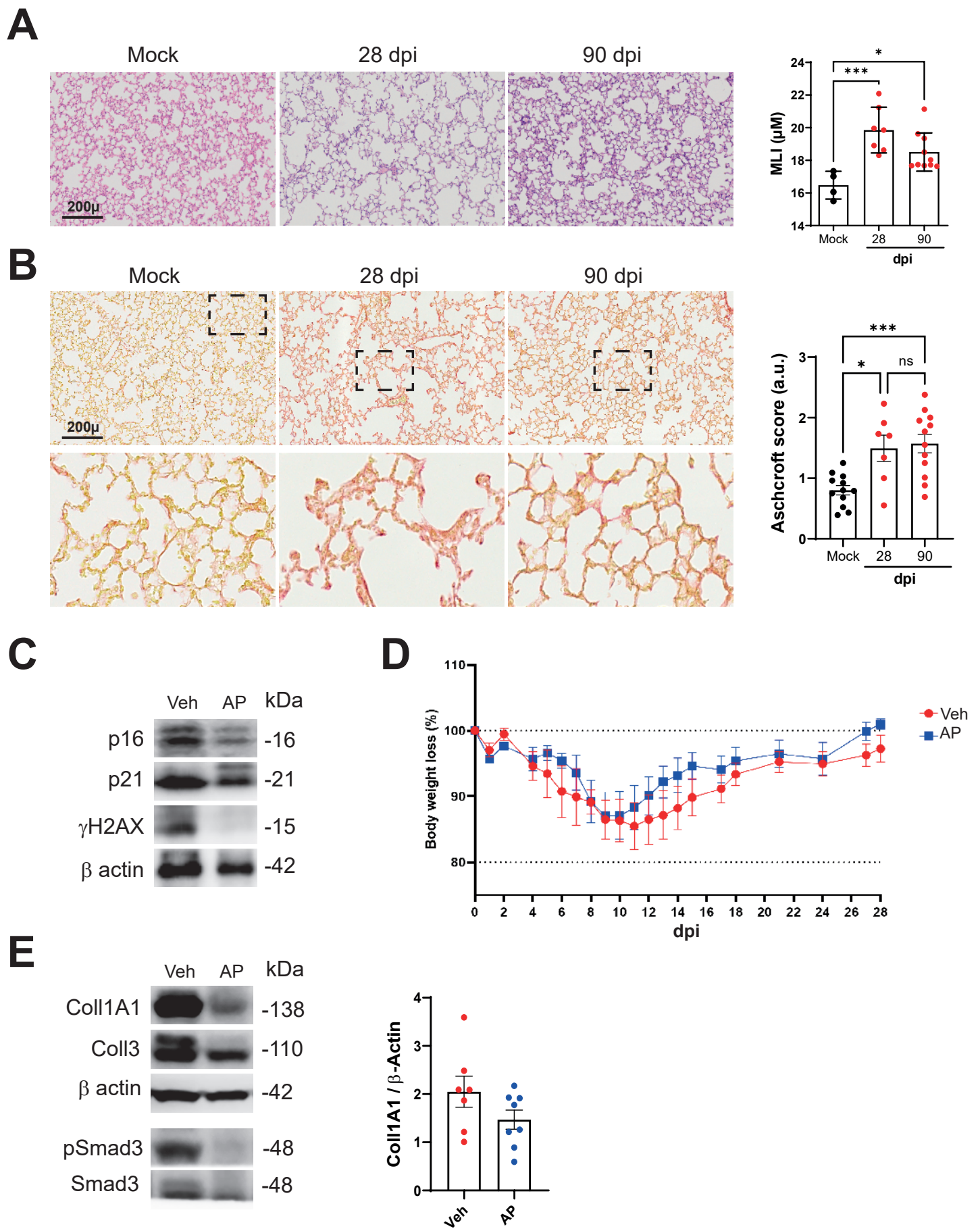

Supplement: Supplementary file 4 — Figure S4. Analysis of lung emphysema and pulmonary fibrosis in IAV‐infected p16‐ATTAC mice. (A, B) Lungs were collected on 28 and 90 dpi. Lung sections were stained with hematoxylin/eosin (A) or Sirius red (B) (bar = 200 μ). Left panels, Representative micrographs. Right panels, scatter plot showing mean linear intercept (MLI) and parenchymal fibrosis quantification. (C) Representative western blotting showing the expression of p16, p21, γ‐H2A.X, and beta actin in vehicle and AP20187‐treated IAV‐infected mice (28 dpi) (whole lung homogenates). (D) Effect of AP20187 treatment on body weight loss and body weight regain during the course of infection (n = 8/group). (E) Left panel, representative western blotting showing the expression of collagen 1 alpha 1 (Coll1A1), collagen 3 (Coll3), and unphosphorylated and phosphorylated Smad3 (whole lung homogenates). Right panel, Scatter‐plot graphs representing the relative expression of (n = 7‐8/group). Significant differences were determined using a one‐way ANOVA followed by Bonferroni post hoc test (A, B) or the two‐tailed Mann–Whitney U‐test (E) (*p < 0.05, ***p < 0.001). [file ACEL-24-e70140-s002.pdf]

Supplemental Figure 5

**A**

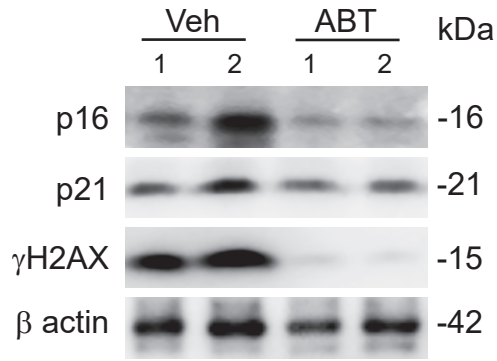

**B**

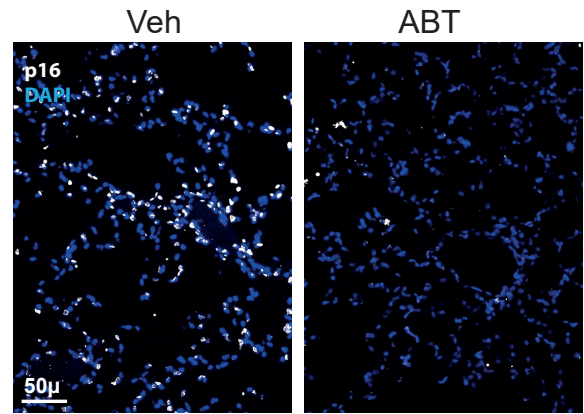

**C**

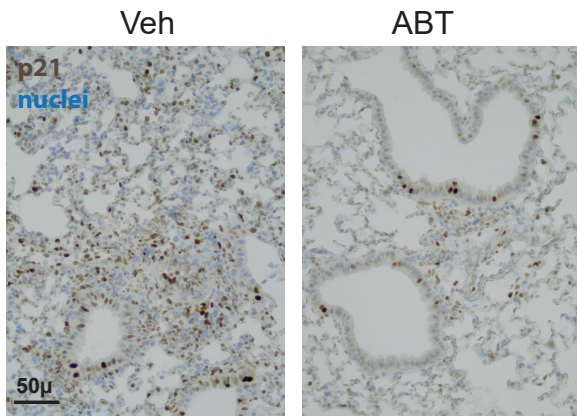

**D**

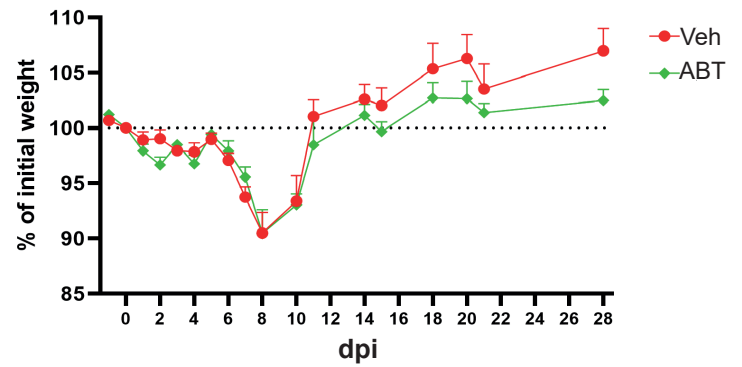

**E**

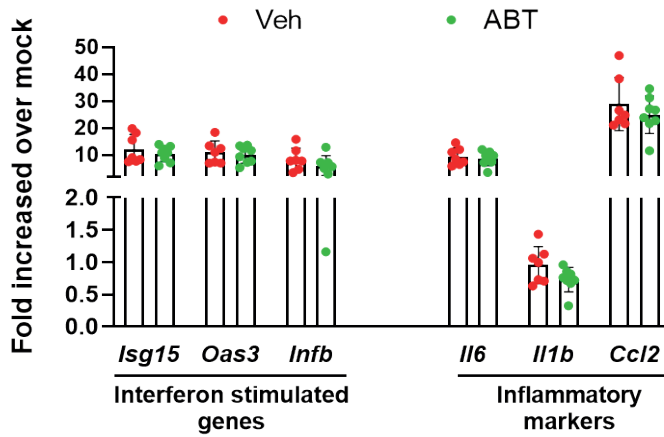

**F**

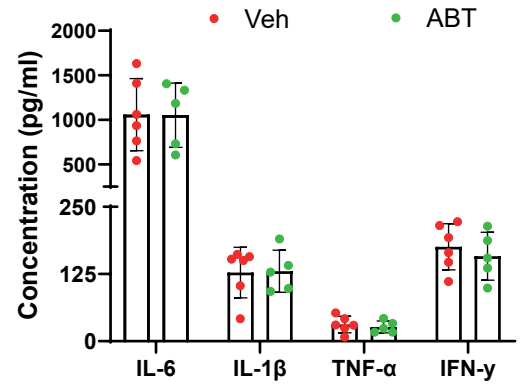

Supplement: Supplementary file 5 — Figure S5. Effect of ABT‐263 on senescent cell marker expression and on influenza outcomes. (A) Western blotting showing the expression of p16, p21, and γH2AX and β‐actin in vehicle‐ and ABT‐263‐treated mice (28 dpi) (whole lung homogenates). (B, C) Elimination of p16‐positive cells (white) and p21‐positive cells (brown) by ABT‐263 as assessed by immunohistochemistry (28 and 7 dpi, respectively). Bar = 50 μ. (D) Effect of ABT‐263 treatment on the kinetics of body weight loss and body weight regain. (E) Expression of ISGs and inflammatory genes by quantitative RT‐PCR (7 dpi). (F) Quantification of inflammatory cytokines in lung extracts. (D–F) n = 7‐8/group. [file ACEL-24-e70140-s004.pdf]
